# *Pseudomonas aeruginosa* Suppresses Host Immunity by Activating the DAF-2 Insulin-Like Signaling Pathway in *Caenorhabditis elegans*

**DOI:** 10.1371/journal.ppat.1000175

**Published:** 2008-10-17

**Authors:** Eric A. Evans, Trupti Kawli, Man-Wah Tan

**Affiliations:** Department of Genetics and Department of Microbiology and Immunology, Stanford University School of Medicine, Stanford, California, United States of America; Massachusetts General Hospital, United States of America

## Abstract

Some pathogens have evolved mechanisms to overcome host immune defenses by inhibiting host defense signaling pathways and suppressing the expression of host defense effectors. We present evidence that *Pseudomonas aeruginosa* is able to suppress the expression of a subset of immune defense genes in the animal host *Caenorhabditis elegans* by activating the DAF-2/DAF-16 insulin-like signaling pathway. The DAF-2/DAF-16 pathway is important for the regulation of many aspects of organismal physiology, including metabolism, stress response, longevity, and immune function. We show that intestinal expression of DAF-16 is required for resistance to *P. aeruginosa* and that the suppression of immune defense genes is dependent on the insulin-like receptor DAF-2 and the FOXO transcription factor DAF-16. By visualizing the subcellular localization of DAF-16::GFP fusion protein in live animals during infection, we show that *P. aeruginosa*–mediated downregulation of a subset of immune genes is associated with the ability to translocate DAF-16 from the nuclei of intestinal cells. Suppression of DAF-16 is mediated by an insulin-like peptide, INS-7, which functions upstream of DAF-2. Both the inhibition of DAF-16 and downregulation of DAF-16–regulated genes, such as *thn-2*, *lys-7*, and *spp-1*, require the *P. aeruginosa* two-component response regulator GacA and the quorum-sensing regulators LasR and RhlR and are not observed during infection with *Salmonella typhimurium* or *Enterococcus faecalis*. Our results reveal a new mechanism by which *P. aeruginosa* suppresses host immune defense.

## Author Summary

Bacterial pathogens have evolved mechanisms to overcome the immune defenses that animals and plants deploy against them. In some cases, this involves directly interfering with the proper functioning of the immune system. Because pathogens that employ these strategies are often the most deadly and difficult to treat, it is important to understand how they are able to suppress the immune system in the context of the whole organism. In this paper, we show that *Pseudomonas aeruginosa*, a bacterial pathogen that is a major contributor to hospital-borne infections such as pneumonia, suppresses an immune defense pathway during infection of the simple animal host *Caenorhabditis elegans*. Using genetic modifications of both the pathogen and host, we identify components of the signaling pathways required to suppress host immune defenses. We find that *P. aeruginosa* employs the cell-to-cell communication system known as quorum sensing, which coordinates the expression of virulence factors to suppress host immune defense. In the host, an evolutionarily conserved insulin-like signaling pathway is affected by *P. aeruginosa*, resulting in the suppression of genes that are required for defense against infection in the intestinal epithelial cells. These findings suggest the possibility that *P. aeruginosa* may exploit similar mechanisms when causing infections of human epithelium, such as the epithelial lining of the lungs.

## Introduction

The innate immune system is a genetically-encoded host defense mechanism that constitutes the first line of defense against pathogens in plants and animals. Innate immunity in animals is evolutionarily ancient, and the molecular components of mammalian innate immunity are partly conserved in invertebrates such as *Drosophila* and *C. elegans* that lack a somatic recombination-based adaptive immunity. Innate immunity comprises several functions, including the production of defense proteins, such as antimicrobial peptides, lysozymes, and other immune modulators [1]. A common theme emerging from the studies of host-pathogen interactions is that recognition of pathogen-associated molecular patterns (PAMPs) or of infection byproducts by host receptors, such as the Toll-like receptors (TLRs), trigger highly regulated immune responses, including the induction of antimicrobial effectors [2],[3]. For example, infection by Gram-positive bacteria triggers activation of the Toll pathway in *Drosophila*. Activation of Toll signaling in turn induces expression of specific antimicrobial genes through the activation of a Rel/NF-κB transcription factor [4],[5]. Despite lacking a functionally conserved TLR/NF-κB pathway [6], *C. elegans* is able to mount robust immune responses against a variety of pathogens (see [7]–[10] for examples). This underscores the importance of other pathways in *C. elegans* innate immunity. Recently, through genetic studies, several conserved signal transduction pathways that are required for innate immunity in *C. elegans* have been identified. They include the p38 MAPK, the Sma/TGF-β, and the DAF-2/DAF-16 insulin-like signaling pathways (reviewed in [11],[12]). For example, mutants in *sek-1*, which encodes a p38 MAPK kinase, and *pmk-1*, which encodes a p38 MAPK, are sensitive to killing by bacterial pathogens [13]. Mutants in *sma-6*, which encodes a TGF-β receptor, are also sensitive to killing by bacterial pathogens [10],[14]. In contrast, mutants in *daf-2*, which encodes an insulin-like receptor, are resistant to killing by bacterial pathogens. The resistance of *daf-2* mutants is completely dependent on DAF-16, a FOXO transcription factor [15]. Microarray studies suggest that each of these immune signaling pathways regulates the expression of host effector genes, which may account for the altered pathogen susceptibility of pathway mutants [8],[16],[17].

Host defense effectors include diverse classes of small molecules and antimicrobial peptides. Lysozyme, a bacteriolytic enzyme, is ubiquitously expressed in mammalian secretions. The *C. elegans* genome encodes ten lysozyme-like proteins (*lys-1* to *lys-10*), some of which have been directly implicated in host defense [7],[10]. The *C. elegans* genome also encodes many amoebapore or saposin-like proteins (e.g., *spp-1*) that are members of a large and diverse class of antimicrobial peptides and a number of defensin-like molecules homologous to ASABF (*Ascaris suum* antibacterial factor), including *abf-2*. In mammals and *Drosophila*, the expression of defensin-family proteins contributes to antibacterial, antifungal, and antiviral defenses. Recombinant SPP-1 and ABF-2 have antimicrobial activity [18],[19], and endogenous expression contributes to antibacterial defense in *C. elegans* [20]. The *C. elegans* genome also encodes homologs of the thaumatin family of plant antifungal proteins (*thn-1* to *thn-8*). While it is not known whether *C. elegans* thaumatins have antifungal activity, RNAi knockdown of *thn*-family genes results in worms that are more sensitive to killing by *P. aeruginosa* [7]. Host effector genes are differentially regulated during infection of *C. elegans* [7]–[10]. Microarray studies also suggest that different pathogens elicit specific transcriptional responses [21]. This is presumably due to host recognition of different PAMPs followed by induction of specific host defense pathways. For example, during fungal infection, TIR-1 (Toll and IL-1 receptor) activates an antifungal defense through p38 MAPK signaling [22]. The mechanisms by which specific transcriptional responses are elicited are still largely unknown. However, the intestine-specific GATA transcription factor ELT-2 is required for the regulation of host immune defense effectors and resistance to bacterial pathogens [7],[23].

*P. aeruginosa* is an important Gram-negative human pathogen that is associated with infection of immunocompromised patients [24], including cystic fibrosis (CF) patients and individuals with burn wounds [25],[26]. *P*. *aeruginosa* has evolved at least three strategies to combat the vast repertoire of host defenses. First, *P. aeruginosa* can produce an extensive array of cell-associated and secreted virulence factors that are deleterious or damaging to the host. A substantial number of these virulence determinants are regulated by the two-component regulator encoded by *gacA* and the quorum-sensing regulators encoded by *lasR* and *rhlR* [27],[28]. For example, *P. aeruginosa* secretes several phenazines, including pyocyanin [29], that have tissue-damaging properties attributed to the induction of free radical production in host cells [30]. Second, *P. aeruginosa* can evade detection by the host, either by directly destroying host molecules that are involved in pathogen detection or by downregulating PAMP expression. For example, an important component of host defense is the deposition of a complement component C3b on the bacterial surface, leading to the induction of host responses and pathogen clearance [31]. To counter complement activation, *P. aeruginosa* produces alginate to limit accessibility of complement and secretes proteases, including alkaline protease and elastase that degrade C3b [32],[33]. The flagellum, an important virulence determinant required for motility and attachment, is also a PAMP that is detected by the host through the interaction of monomeric flagellin with TLR5, resulting in NF-κB and p38 MAPK activation; this innate immune response is intended to protect the host. Upon growth on purulent mucus from CF and non-CF patients, *P. aeruginosa* downregulates flagellin synthesis, thereby blunting the host protective immune response [34],[35]. A third strategy is to compromise the host by suppressing the host defense responses. It has been suggested that the ability of *P. aeruginosa* to rapidly kill *Drosophila* is associated with downregulation of antimicrobial peptide expression by a yet-unknown mechanism that requires a putative S-adenosyl-methionine-dependent methyltransfrease [36].

We found that an unexpected feature of the transcriptional response to *P. aeruginosa* is the downregulation of several intestinally-expressed host defense effectors [7],[37]. We hypothesized that repression of immune effector expression, such as *thn-2, spp-1*, and *lys-7*, may represent a virulence mechanism used by *P. aeruginosa* to suppress host defenses. In this report, we identify both host and pathogen factors required for the downregulation of immune effectors during *P. aeruginosa* infection of *C. elegans*. We present data indicating that *P. aeruginosa* infection causes the activation of DAF-2 insulin-like signaling, which leads to translocation of DAF-16 protein from nuclei of intestinal cells and downregulation of DAF-16 transcriptional targets. Delocalization of DAF-16 requires DAF-2 and an upstream neuroendocrine signaling pathway, including the DAF-2 agonist INS-7. Each of these effects is dependent on the *P. aeruginosa* two-component regulator GacA and the quorum-sensing regulators LasR and RhlR. Our results demonstrate that *P. aeruginosa* infection of *C. elegans* results in the suppression of intestinal immune defense through virulence factor mediated effects on the activity of insulin-like signaling in the intestine.

## Results

### *P. aeruginosa* downregulates the expression of host effector molecules

**Figure 1:**
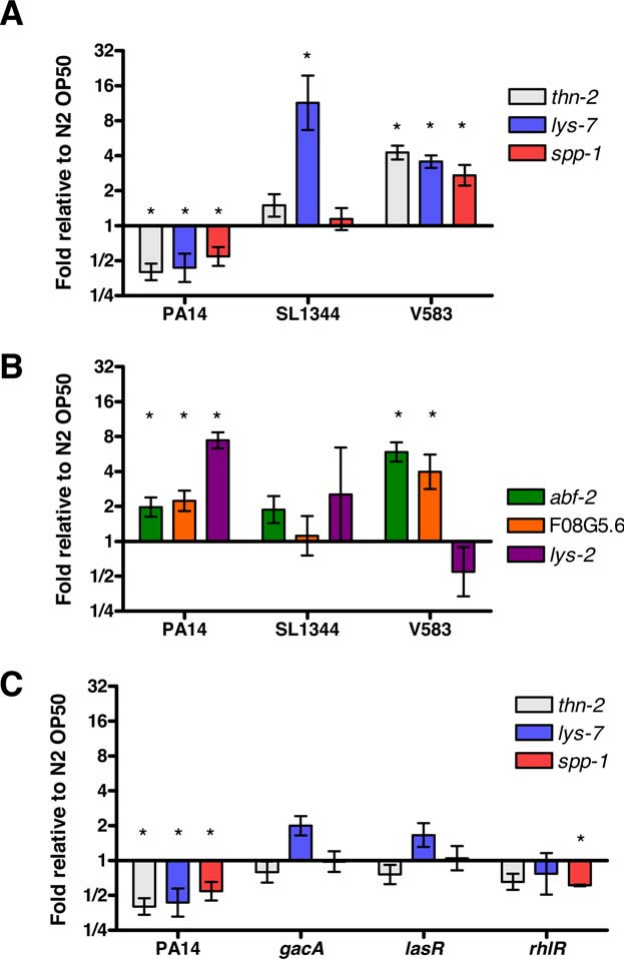
Specific downregulation of host immune effector gene expression following *P. aeruginosa* infection requires *gacA*, *lasR* and *rhlR*. (A–B) Expression of the host defense effector genes *thn-2*, *lys-7*, *spp-1*, *abf-2*, F08G5.6, and *lys-2* in wildtype worms exposed to *P. aeruginosa* PA14, *S. typhimurium* SL1344, and *E. faecalis* V583. (C) Expression of downregulated host defense effectors genes in wildtype worms exposed to the PA14 mutants *gacA*, *lasR*, and *rhlR*. Mean transcript levels are plotted relative to matched controls exposed to OP50-1. Error bars indicated SEM. At least 3 replicates of each condition were examined. * t-test, p<0.05 comparison to N2 on OP50-1.

An important component of the innate immune response in plants and animals is the induced expression of host defense effectors following pathogenic challenge [38]. Several genes that are induced following infection and are important to protect *C. elegans* from pathogenic challenge have been identified. These host defense effectors include defensin (*abf-2*), saposin (*spp-1*), and several genes with homology to antimicrobial proteins: lysozymes (*lys-2* and *lys-7*), thaumatin (*thn-2*), and a CUB-domain containing protein, F08G5.6. The expression of *abf-2* and *spp-1* was induced following infection by the Gram-negative bacterium *Salmonella typhimurium*, and both genes were required to protect against *S. typhimurium* infection [20]. Independent microarray studies showed that *lys-2* and F08G5.6 were induced following infection by *P. aeruginosa* and that *thn-2* and *lys-7* were induced by the Gram-positive pathogens *E. faecalis* and *M. nematophilum* [7]–[9],[21]. Interestingly, *spp-1*, *thn-2*, and *lys-7* expression was repressed during infection by *P. aeruginosa* [7],[8],[39]. This expression pattern is unexpected given that these genes are required for optimal survival on *P. aeruginosa* (Figure S1). To confirm the expression data, we used quantitative RT-PCR (qRT-PCR) to measure the expression of these genes in worms exposed to *P. aeruginosa* (PA14), *E. faecalis* (V583), and *S. typhimurium* (SL1344) for 12 hours at 25°C (Figure 1A–B). Three of the genes (*thn-2*, *lys-7*, and *spp-1*) were significantly repressed by exposure to *P. aeruginosa* compared to the laboratory food source *E. coli* (OP50-1) (Figure 1A). Three of the genes (*abf-2*, F08G5.6, and *lys-2*) were significantly induced by exposure to *P. aeruginosa* (Figure 1B). In contrast to *P. aeruginosa*, none of the six genes tested were significantly repressed following exposure to either *S. typhimurium* or *E. faecalis*. Among the genes that were significantly repressed by exposure to *P. aeruginosa*, *lys-7* was induced by exposure to *S. typhimurium* and *E. faecalis*, while *thn-2* and *spp-1* were induced by exposure to *E. faecalis* (Figure 1A). Though we did not detect a significant induction of *spp-1* by *S. typhimurium* following 12-hour exposure (Figure 1A), *spp-1* has been previously observed to be induced after 48-hour exposure to *S. typhimurium* [20]. Thus, the downregulation of known immune effectors appears to be an atypical response to infection that is characteristic of infection by *P. aeruginosa* because similar responses are not observed by exposure to *S. typhimurium* or *E. faecalis*.

### Downregulation of effector genes requires *P. aeruginosa* virulence regulators

*P. aeruginosa* GacA is a two-component response regulator that is required for full virulence in *C. elegans* and mammals [40]–[42]. GacA regulates the production of molecules that are detrimental to the host, including pyocyanin, elastase, and exotoxin A [27],[28]. We wondered whether the suppression of host defense effectors is an active outcome of infection: specifically, whether GacA regulates factors that are important in downregulating host defense gene expression in *C. elegans*. We therefore compared the expression of *thn-2*, *lys-7*, and *spp-1* in worms exposed to the *P. aeruginosa gacA* mutant for 12 hours (Figure 1C). In contrast to wildtype *P. aeruginosa* (hereafter referred to as PA14), none of the genes were repressed by exposure to an in-frame deletion mutant of *gacA* (PA14 *gacA*). This indicates that downregulation of immune genes by PA14 requires *gacA*. The quorum-sensing regulators encoded by *lasR* and *rhlR* function downstream of GacA [27]. *lasR* and *rhlR* are also required for PA14 virulence in *C. elegans* ([42] and Table S1). As with exposure to PA14 *gacA*, *thn-2*, *lys-7*, and *spp-1* were not repressed in worms exposed to a *lasR* transposon insertion mutant (PA14*lasR*) compared to OP50-1 (Figure 1C). In worms exposed to a *rhlR* transposon-insertion mutant (PA14*rhlR*), neither *thn-2* nor *lys-7* was downregulated, but *spp-1* remained significantly repressed (Figure 1C). Thus, the ability of PA14 infection to downregulate the expression of *thn-2* and *lys-7* requires factors that are dependent on *gacA*, *lasR*, and *rhlR*. By contrast, while the downregulation of *spp-1* requires *gacA* and *lasR*, it is independent of *rhlR*.

The PA14 *gacA* and PA14 *lasR* mutant strains are unable to colonize the worm intestine [42]. To determine whether the failure of PA14 *gacA*, PA14 *lasR*, and PA14 *rhlR* mutants to downregulate immune gene expression is a consequence of a low inoculum in the intestine, we compared the expression of *thn-2*, *lys-7*, and *spp-1* in the *C. elegans tnt-3(aj3)* mutant following infection with the wildtype PA14, PA14 *gacA*, PA14 *lasR*, and PA14 *rhlR* strains. The *tnt-3(aj3)* mutant is unable to grind bacteria, thus a large inoculum of live bacteria, including the nonpathogenic OP50-1, accumulate in the intestinal lumen [13],[20]. First, we observed that although the *tnt-3(aj3)* animals accumulate OP50-1 in the intestine, expression of *thn-2*, *lys-7*, and *spp-1* was indistinguishable from expression in wildtype worms which do not accumulate OP50-1 (t-test, p = 0.46, 0.43, and 0.07, respectively). Importantly, despite the accumulation of intact PA14 *gacA*, PA14 *lasR*, or PA14 *rhlR* mutant bacteria within the intestinal lumen of *tnt-3(aj3)* animals, the expression of *thn-2, lys-7, and spp-1* was not repressed (Figure S2). Thus, the lack of immune suppression by the PA14 *gacA*, PA14 *lasR*, and PA14 *rhlR* mutants is not due simply to limited intestinal colonization of wildtype worms. These results, coupled with the observation that *S. typhimurium* and *E. faecalis* infections do not downregulate *thn-2*, *lys-7*, and *spp-1* expression (Figure 1A), indicate that the mere presence of live bacteria in the intestinal lumen is insufficient to suppress the expression of a subset of immune genes. We conclude that a genetically regulated aspect of PA14 virulence, which is absent in *S. typhimurium* or *E. faecalis*, causes downregulation of a subset of immune effectors in *C. elegans*.

Several Gram-negative bacterial pathogens suppress host immunity by employing the type III secretion system (T3SS), which injects virulence effectors directly into host cytoplasm to inhibit or limit the duration of NF-κB and MAP kinase activation (reviewed in [43],[44]). In *P. aeruginosa*, T3SS is an important virulence determinant in pathogenesis in insects and mammals [45]–[48]. Type III secretion genes are under the control of the GacS/GacA two-component regulator and the quorum-sensing system [49],[50]. To date, four T3SS effector proteins have been identified for *P. aeruginosa*: ExoS, ExoT, ExoU, and ExoY. In PA14, only ExoT, ExoU, and ExoY are encoded in the genome and their expression requires PscD. Although the T3SS is induced during infection of the *C. elegans* intestine, it appears to be dispensable for *C. elegans* killing [46],[51]. This led to the proposal that other virulence factors that play a predominant role in *C. elegans* pathogenesis could mask the effect of type III secretion effectors when death was used as the metric for the pathogenesis assay. We therefore determined whether the T3SS contributes to the suppression of *C. elegans* immune effectors, an arguably more sensitive assay. As previously observed, neither loss of any of the Type III effectors nor the entire repertoire of effectors in the *ΔpscD* mutant significantly affects the ability of these strains to kill *C. elegans* ([46] and data not shown). We found that the *ΔpscD* mutant was still able to significantly suppress the expression of *lys-7* and *spp-1*, as measured by qRT-PCR (Figure S3A). We further confirmed that the *P. aeruginosa* T3SS is not required for immune suppression by showing that neither *ΔexoT, ΔexoU, ΔexoY*, nor *ΔpscD* mutant significantly affected the expression of a *lys-7* GFP-reporter (Figure S3B).

In *Drosophila*, downregulation of NF-κB-regulated antimicrobial peptide expression by *P. aeruginosa* is mediated by an unknown mechanism that requires an S-adenosyl-methionine-dependent methyltransferase domain-containing protein encoded by PA14_41070 [36]. A PA14_41070 transposon insertion mutant is not defective in the ability to downregulate *lys-7* or *spp-1* expression, as measured by qRT-PCR (Figure S3A) and by GFP-reporter gene expression of *lys-7* (Figure S3B). Thus, PA14_41070 is dispensable for immune suppression in *C. elegans*.

To determine whether suppression of host defense gene expression is strictly associated with *P. aeruginosa* virulence, we tested several mutants that are impaired in *C. elegans* killing. First, we tested two genes that are part of the GacA-LasR-RhlR regulon: *dsbA* and *pqsA*. Expression of *dsbA*, which encodes a periplasmic dithiol:disulfide oxidoreductase, requires GacA [52]. DsbA is required for the formation of disulfide bonds in periplasmic proteins and important for proper folding of multiple virulence factors exported by Type II secretion, including elastase and lipase. *Pseudomonas* quinolone signal (PQS) system is the third component of the quorum-sensing signaling system, and it is regulated by Las and Rhl quorum-sensing systems [53],[54]. Production of all known quinolone/quinolines in *P. aeruginosa* requires PqsA, an anthranilate-coenzyme A ligase that is the product of the *pqsABCDE* operon [55]–[57]. Both the *dsbA* and *pqsA* mutant strains are attenuated for killing *C. elegans* ([42] and Table S1). Yet, neither the *dsbA* nor the *pqsA* mutants is defective in the ability to downregulate *lys-7* expression, as measured by a GFP-reporter (Figure S3B). Thus, PQS and DsbA, despite their requirement for virulence are not necessary for host immune suppression in the *C. elegans* model. Because *dsbA* and *pqsA* are part of the GacA-LasR-RhlR regulon, these results suggest that only a subset of the genes regulated by the GacA two-component and acyl-homoserine lactone quorum-sensing systems are required for immune suppression.

To determine the specificity of the GacA-LasR-RhlR regulon in immune suppression, we tested three additional genes that are not known to be part of this regulon: PA14_23420, PA14_23430, and PA14_59010 [58],[59]. We found that while these mutants are attenuated for killing *C. elegans* (Table S1), they had no detectable defect in the ability to downregulate *lys-7* expression as measured by a GFP-reporter (Figure S3B). Overall these results indicate that virulence in *P. aeruginosa* is not strictly associated with immune suppression and that a subset of the GacA-, LasR- and RhlR-dependent factors are required for the downregulation of immune genes by *P. aeruginosa*.

### *P. aeruginosa* downregulates a subset of DAF-2–dependent immune genes

In *C. elegans*, at least three conserved signaling pathways contribute to host defense: p38 MAPK signaling, Sma/TGF-β signaling, and DAF-2 insulin-like signaling (reviewed in [11],[12]). We had previously noted that the transcriptional profiles of PA14-infected and *daf-2* loss-of-function worms were overlapping, but the genes regulated in common tend to be regulated in opposite directions [7]. Here, we compared whole-genome transcriptional profiles of uninfected *daf-2* mutants [16],[60] to PA14-infected wildtype animals [7],[8] and found that *daf-2* mutants and PA14 infection have predominately discordant effects on gene expression across a range of data sets using a variety of criteria for comparison (Table S6, Table S7, Figure S10A). This suggests that the gene expression pattern in PA14-infected animals is opposite of the pattern produced by reducing the activity of DAF-2, such as occurs in loss-of-function *daf-2* mutants. Importantly, genes that were inversely regulated by *daf-2* and PA14 infection were enriched for immune effector genes, thus raising the possibility that PA14 infection activates DAF-2 insulin-like signaling (Table S8, Figure S10B; for details, see Text S1).

**Figure 2:**
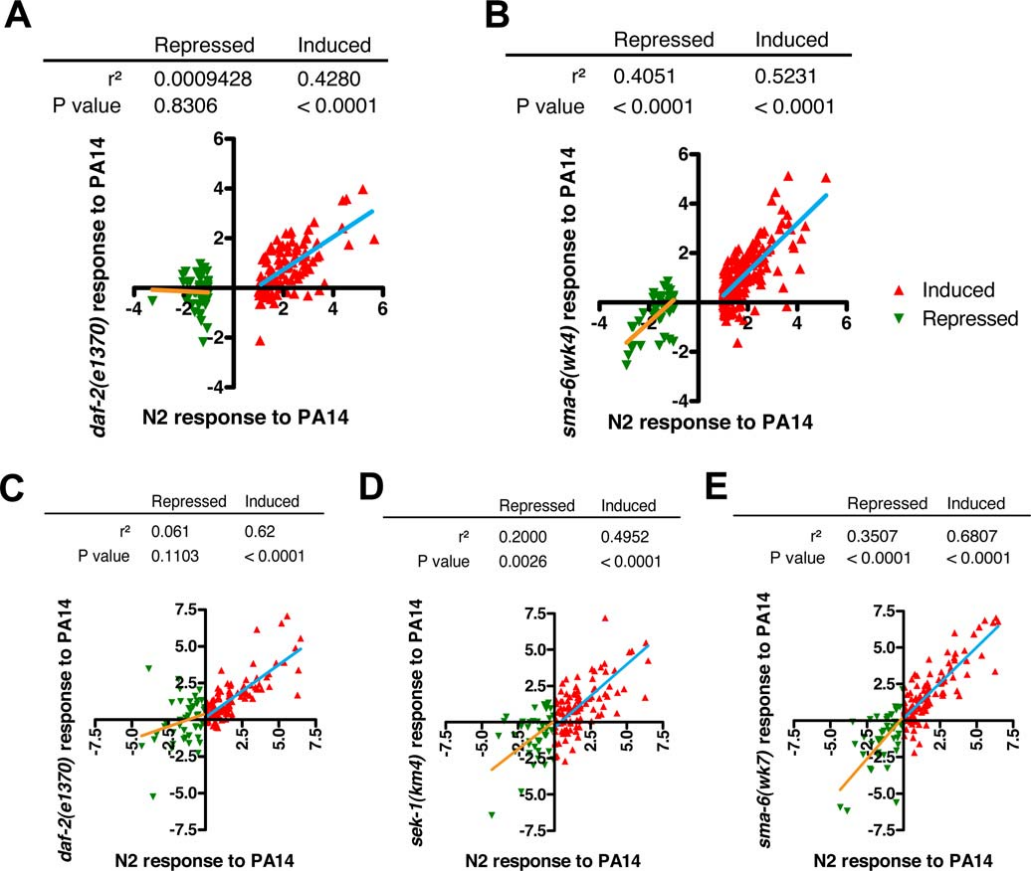
The downregulation of host defense genes is specifically attenuated in *daf-2* insulin-like signaling mutants. (A–B) The transcriptional response to PA14 infection in wildtype N2 versus (A) *daf-2(e1370)* and (B) *sma-6(wk7)* worms is plotted based on whole-genome microarray analysis. Values are log_2_ scale transformations of the response to infection. The subset of genes that were differentially regulated in N2 worms is shown (p<0.05 and absolute difference >2 fold). Linear regressions are plotted for induced (red) and repressed (green) genes independently. (A) In *daf-2(e1370)*, the response to induced genes to PA14 infection was largely intact (r^2^ = 0.4280, p<0.0001), but the response to repressed genes was strongly attenuated (r^2^ = 0.0009, p = 0.8306). (B) In *sma-6(wk7)*, the response of both induced and repressed genes was largely intact (p<0.0001). (C–E) The transcriptional response to PA14 infection in wildtype N2 and (A) *daf-2(e1370)*, (B) *sek-1(km4)*, and (C) *sma-6(wk7)* animals is plotted for a set of 146 candidate immune and stress response genes based on qRT-PCR measurement. Linear regressions were calculated for induced (red) and repressed (green) genes independently. The associated correlation coefficients (r^2^) and p-values are indicated. (C) In *daf-2(e1370)*, the response to induced genes to PA14 infection was largely intact (r^2^ = 0.62, p<0.0001), but the response to repressed genes was strongly attenuated (r^2^ = 0.061, p = 0.1103). (D) In *sek-1(km4)* the response of both induced and repressed genes was largely intact (p<0.0001 and p = 0.0026, respectively). (E) In *sma-6(wk7)* the response of both induced and repressed genes was largely intact (p<0.0001 each).

If indeed activation of DAF-2 insulin-like signaling mediates PA14 suppression of host defense effectors, then the ability of PA14 to suppress host defense effectors would be attenuated or eliminated when loss-of-function *daf-2(e1370)* mutants are infected with PA14. Alternatively, if the transcriptional effect of *daf-2* loss of function and PA14 infection operate in parallel, then the transcriptional response to PA14 will be unaffected in *daf-2(e1370)* animals. We therefore compared the change in gene expression following PA14 infection between wildtype and *daf-2(e1370)* animals using whole-genome microarrays. First, using the criteria of t-test p-values<0.05 and 2-fold up- or down-regulation, we identified 247 induced and 137 repressed genes in wildtype worms infected with PA14 for 24 hours compared to uninfected controls. We confirmed that the selection of these criteria did not affect our conclusions by repeating the analysis with a range of p-value and fold-change thresholds (data not shown). Next, using this list of PA14-induced and -repressed genes, we compared the change in gene expression in response to PA14 in *daf-2(e1370)* and wildtype (N2) animals (Figure 2A). Separate linear regressions of induced and repressed genes revealed that the induction response to PA14 was largely intact in *daf-2(e1370)* animals (r^2^ = 0.4280, p<0.0001), but the repression response to PA14 was substantially attenuated to the extent that the repression response observed in N2 and *daf-2(e1370)* was not significantly associated (r^2^ = 0.001, p = 0.3). Comparison of induced and repressed genes provides an internal control for this analysis and indicates that attenuation of the downregulation of genes in response to PA14 infection does not simply reflect a failure of *daf-2* animals to become infected by PA14 or to respond transcriptionally to PA14 infection. These results are consistent with the model that PA14 infection suppresses host defense genes through activation of the DAF-2 insulin-like signaling pathway. It also indicates that the induction of genes in response to PA14 infection is largely independent of DAF-2.

To examine whether the Sma/TGF-β signaling pathway could also contribute to the suppression of host defense genes following PA14 infection, we compared whole-genome expression data from infected and uninfected TGF-β receptor null mutant *sma-6(wk7)* and wildtype worms. Using the same parameters as the *daf-2(e1370)* analysis, we note that both the induction and repression of genes in response to PA14 infection were largely intact in *sma-6(wk7)* (r^2^ = 0.5231 and 0.4051, respectively, p<0.0001 each; Figure 2B). The correlation coefficients for induced and repressed genes were not significantly different. Comparison of the regression results from *daf-2(e1370)* with *sma-6(wk7)* is revealing. The correlations for induced genes between the *daf-2(e1370)* and *sma-6(wk7)* analyses (r^2^ = 0.4280 and r^2^ = 0.5231, respectively) were not significantly different (p = 0.09, one-tailed test). Among the repressed genes, however, correlations with *daf-2(e1370)* were significantly less than with *sma-6(wk7)* (p = 10^−5^, one-tailed test). Together, the data indicate that the Sma/TGF-β pathway is unlikely to contribute substantially to the suppression of host defense genes by PA14 infection.

To corroborate the results obtained from whole-genome microarray analysis, and also to analyze the involvement of p38 MAPK signaling, we repeated the transcriptional profile analysis using qRT-PCR measurement of a panel of 146 infection and stress response genes. We designed gene-specific qRT-PCR primers to a panel of 146 genes selected on the basis of their likely involvement in the response to infection and stress. Many immune and stress response genes are members of large gene families [7],[61]. The use of gene-specific qRT-PCR primers overcomes the problem of cross hybridization of gene families in microarray studies and provides more precise measures of mRNA levels. Moreover, the use of genes with known or putative function in immune and stress response supports the inference that observed transcriptional effects are functionally important. We measured gene expression in young adult worms exposed to OP50-1 or PA14 for 12 hours for N2, *daf-2(e1370)*, *sma-6(wk7)*, and *sek-1(km4)*, a p38 MAPKK null mutant, to obtain the change in gene expression following infection for each worm strain (Table S2). We repeated the linear regression analysis of induced and repressed genes, comparing the transcriptional response to PA14 in each mutant to N2 (Figure 2C–2E). Transcriptional analysis of *daf-2(e1370)* using this targeted gene set indicated that the induction of immune and stress response genes in response to PA14 was largely intact in *daf-2(e1370)* animals (r^2^ = 0.62, p<0.0001), but the repression of genes in response to PA14 was substantially attenuated to the extent that the correlation between N2 and *daf-2(e1370)* was not statistically significant (r^2^ = 0.061, p = 0.11; Figure 2C). This pattern of gene expression mirrors the result of the whole-genome analysis (Figure 2A) and provides further support that a substantial subset of the normal downregulation of gene expression in response to PA14 infection requires *daf-2*, but the upregulation of gene expression is largely independent of *daf-2*. In contrast to *daf-2(e1370)*, both induced and repressed genes in *sek-1(km4)* and *sma-6(wk7)* mutants correlated significantly with N2 (Figure 2D–2E), thus corroborating the results of our whole-genome analysis with *sma-6(wk7)*. Visually, the induced and repressed linear regression lines are concordant for *sek-1(km4)* (Figure 2D) and *sma-6(wk7)* (Figure 2E), but highly discordant in *daf-2(e1370)* (Figure 2C). Analysis with a p38 MAPK mutant *pmk-1(km25)* yielded nearly identical results to *sek-1(km4)*: the response to PA14 infection in *pmk-1(km4)* correlated significantly (r^2^ = 0.80, p<0.0001) with the response in *sek-1(km4)* (data not shown).

To detect more subtle effects of *sma-6, sek-1* and *daf-2* mutations on transcriptional responses to PA14 we compared the average differential expression of the immune and stress response genes under normal (OP50-1 exposure) and infection (PA14) conditions between mutant (X_mutant_) and wildtype (X_wildtype_) worms using a paired t-test (see Materials and Methods). The average difference in induction or repression (X_Δ_ = X_mutant_−X_wildtype_) was calculated for each mutant-wildtype pair. This approach accounts for both the magnitude and direction of attenuation of the response to infection, with positive values of X_Δ_ indicating attenuated repression and negative values of X_Δ_ indicating attenuated induction.

In *sma-6(wk7)* mutants, the correlation analysis indicated that both induction and repression were largely intact. Consistent with this analysis, the average difference in either induction or repression (X_Δ_) of immune and stress response genes in *sma-6(wk7)* mutants was not significantly different from wildtype (induction: X_Δ_ = 0.09, p = 0.38; repression: X_Δ_ = 0.00, p = 0.99). It remains possible that the increased susceptibility of *sma-6(wk)* to PA14 may be a consequence of deregulation of immune gene expression that could not be detected by this analysis.

In *sek-1(km4)* mutants, the average induction in response to infection is significantly less than that of wildtype (induction: X_Δ_ = −0.61, p<10^−4^), indicating that the induction response is attenuated when p38 signaling is abrogated. Although there is a trend towards attenuation of host gene repression in *sek-1(km4)*, the overall effect is not statistically significant for this set of 42 genes (X_Δ_ = 0.38, p = 0.16). Thus, a definitive conclusion regarding the requirement of p38 in repression of immune genes following PA14 infection awaits a whole-genome analysis. By this analysis we are able to show that p38 MAPK signaling is required for the induction of many genes during PA14 infection, consistent with a previous report that p38 MAPK is an important regulator of the transcriptional response to PA14 [8]. Immune and stress response genes that require *sek-1* for induction by PA14 include *clec-85*, *lys-1*, *lys-8*, F35E12.5, Y40D12A.2 and *gst-38*. Interestingly, with the exception of *gst-38*, the basal expression of these genes under normal growth conditions on OP50-1 also requires *sek-1* (Figure S4A–S4F). We also identified a class of genes whose expression levels during normal growth and following infection require *sek-1* but whose induction or repression in response to PA14 do not require *sek-1*; they include *lys-2*, *cpr-3*, *spp-18*, F55G11.2, and T10D3.6 (Figure S4G–S4K). For example, expression levels of *lys-2* in *sek-1(km4)* animals are 0.5% of levels in wildtype worms both in worms exposed to OP50-1 and worms exposed to PA14, yet expression of *lys-2* is induced by a similar ratio in wildtype and *sek-1* mutant worms (Figure S4G).

The average differences in induction and repression of immune and stress response genes in *daf-2(e1370)* mutants were both significantly different from wildtype (induction: X_Δ_ = −0.36, p = 0.0004; repression: X_Δ_ = 1.32, p<10^−5^). Thus, by this analysis we are able to detect a small but significant attenuation in gene induction following infection in *daf-2(e1370)* mutants, suggesting that *daf-2* activity is required for the induction of a small subset of infection-responsive genes, which include *abf-2*, *lys-2*, F08G5.6, ZK6.11, and C17H12.8 (Table S2). The effect of *daf-2(e1370)* on repression of gene expression in response to infection was the strongest observed effect, consistent with the regression analysis. The immune and stress response genes that require *daf-2* for repression include *thn-2*, *lys-7 spp-1*, and *gst-4* (Table S2). Importantly, among *sma-6(wk7), sek-1(km4)*, and *daf-2(e1370)*, only *daf-2(e1370)* significantly attenuated repression of infection response genes. Together, the transcriptional analyses confirm that of the three immune signaling pathways, DAF-2 insulin-like signaling is required for the downregulation of many genes during PA14 infection.

### Suppression of some host defense genes is DAF-16–dependent

**Figure 3:**
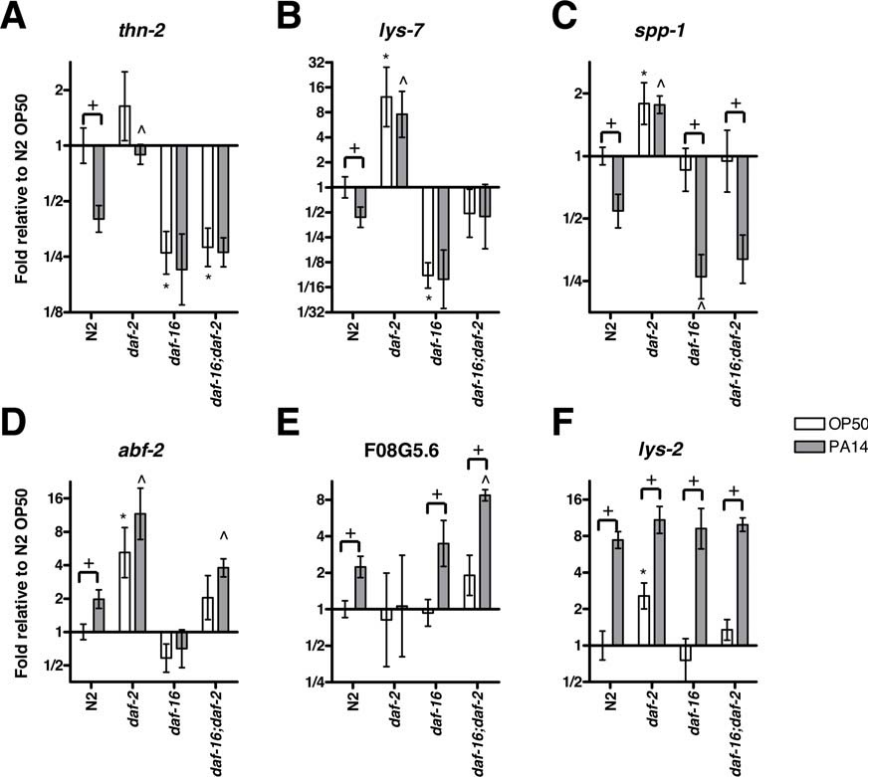
DAF-2/DAF-16 insulin-like signaling mediates the downregulation of host defense effector gene expression by *P. aeruginosa*. Expression of the host defense effectors (A) *thn-2*, (B) *lys-7*, (C) *spp-1*, (D) *abf-2*, (E) F08G5.6, and (F) *lys-2* were measured in N2, *daf-2(e1370)*, *daf-16(mu86)*, and *daf-16(mu86)*;*daf-2(e1370)* exposed to OP50-1 and PA14. Mean transcript levels from at least 3 independent experiments were plotted relative to N2 exposed to OP50-1. Error bars represent SEM. t-test * p<0.05 comparison to N2 OP50-1, ˆ p<0.05 comparison to N2 PA14, + p<0.05 comparing OP50-1 to PA14.

Loss-of-function *daf-2* mutants are resistant to bacterial pathogens, and this resistance is dependent on the FOXO transcription factor DAF-16 [15]. DAF-2 regulates DAF-16 at least in part through the activation of phosphoinositide 3-kinase (PI3-kinase), which is encoded by *age-1* [62],[63]. PI3-kinase potentiates the activity of several serine threonine kinases, including homologs of mammalian PDK1, AKT, and SGK [64]–[66]. These kinases phosphorylate DAF-16, retaining it in the cytoplasm and suppressing DAF-16 transcriptional activity [67],[68]. To determine whether DAF-16 is required for the suppression of host defense genes, we quantified the mRNA levels of *thn-2*, *lys-7*, and *spp-1* by qRT-PCR in N2, *daf-2(e1370)*, *daf-16(mu86)*, and double mutant *daf-16(mu86)*;*daf-2(e1370)* worms under normal growth conditions (on OP50-1) or following infection with PA14. Downregulation of *thn-2*, *lys-7*, and *spp-1* by PA14 exposure was abolished in *daf-2(e1370)* (Figure 3A–3C), confirming that *daf-2* activity is required for the downregulation of a number of functionally important immune genes. Next, we inactivated each of these genes by RNAi in *daf-2(e1370)* and compared the degree of colonization by a PA14 strain that expresses GFP (PA14-GFP) in these animals to *daf-2(e1370)* animals exposed to vector control. Knockdown of *thn-2*, *lys-7,* and *spp-1* individually resulted in a significant increased in colonization (Figure S5), indicating that the ability to prevent downregulation of immune genes contributes to the resistance of *daf-2(e1370)* to PA14. Under normal growth conditions, both *thn-2* and *lys-7* were expressed at lower levels in *daf-16(mu86)* compared to N2, indicating that *daf-16* is required for basal expression of these immune effectors (Figure 3A–3B). Following infection with PA14, the levels of *thn-2* and *lys-7* mRNA were not reduced further in *daf-16(mu86)*, indicating that suppression of *thn-2* and *lys-7* by PA14 requires *daf-16* (Figure 3A–3B). Curiously, the expression of *lys-7* in *daf-16(mu86)*;*daf-2(e1370)* double mutants was intermediate to the expression in either single mutant. Nonetheless, *lys-7* expression was not repressed by PA14 infection in either single or double mutants. These results suggest that basal levels of *lys-7* expression are regulated by *daf-2*-dependent factors in addition to DAF-16. By contrast, under normal growth conditions, *spp-1* expression was not significantly different between N2 and *daf-16(mu86)*, indicating that the basal expression of *spp-1* is independent of *daf-16* (Figure 3C), in contrast to previous reports [39]. Curiously, as in wildtype animals, *spp-1* expression was significantly downregulated in *daf-16(mu86)* and *daf-16(mu86);daf-2(e1370)* mutants, indicating that while the downregulation of *spp-1* is *daf-2*-dependent, it does not require *daf-16*. Overall, these results implicate DAF-2 and DAF-16 in the downregulation of *thn-2*, *lys-7*, and *spp-1* during PA14 infection. DAF-16 appears to be required for the expression of *thn-2* and *lys-7*, but the role of DAF-16 in the regulation of *spp-1* expression is more complex.

Next, we determined whether the induction of *abf-2*, F08G5.6, and *lys-2* by PA14 infection is mediated by *daf-2* and *daf-16* (Figure 3D–3F). In contrast to wildtype, the expression of *abf-2* was not significantly induced in *daf-2(e1370)*, *daf-16(mu86)*, and *daf-16(mu86);daf-2(e1370)* mutants following infection, suggesting that the induction of *abf-2* is modulated by the DAF-2/DAF-16 signaling pathway (Figure 3D). The induction of F08G5.6 was not statistically significant only in *daf-2(e1370)*, suggesting that F08G5.6 induction requires *daf-2* but not *daf-16* (Figure 3E). The expression of F08G5.6 in *daf-2* and *daf-16* single and double mutants mirrors the expression pattern of *spp-1*. Thus, the induction of some genes is dependent on *daf-2* as suggested by the paired t-test analysis performed on the immune and stress response gene set. Expression of *lys-2* was largely unaffected in the insulin-like signaling mutants. However, expression of *lys-2* was higher in *daf-2(e1370)* under basal conditions (on OP50-1). This difference was not observed in worms exposed to PA14, resulting in net a reduced induction of *lys-2* in *daf-2(e1370)* (Figure 3F). Overall, the gene expression analyses suggest that while DAF-2 and DAF-16 are largely dispensable for the induced response to PA14 infection, some genes do require DAF-2 for induction.

### *Pseudomonas* infection suppresses DAF-2–regulated stress response genes

In addition to immune effectors, the insulin-like signaling pathway also regulates stress response genes [16],[69]. For example, *sod-3* encodes a well-characterized DAF-16-regulated superoxide dismutase that is associated with oxidative stress resistance and longevity in *C. elegans*, presumably through its reactive oxygen species detoxification activity [16],[70],[71]. We showed by qRT-PCR that the expression of *sod-3* was significantly repressed following 24-hour exposure to PA14 (Figure S6), consistent with previous microarray studies [8]. This further supports the model that PA14 activates DAF-2 and inhibits DAF-16. Suppression of an antioxidant may be beneficial to *P. aeruginosa*, which produces pyocyanin as a virulence determinant that causes oxidative damage to host tissues [30]. However, knockdown of *sod-3* by RNAi in *daf-2* animals did not significantly affect the ability of *daf-2* mutants to resist colonization by PA14 (Figure S5), perhaps because of the expression of other genes that contributes to oxidative stress resistance, such as *gst-4*, *mtl-1*, and *ctl-1*, are also increased in *daf-2* mutants [16]. We note that the expression of *gst-4*, which encodes a glutathione-S transferase [72] is also downregulated during PA14 infection, as determined by reporter gene expression (Figure S7A) and qRT-PCR analyses (Figure S7B). Downregulation of *gst-4* by PA14 requires *gacA*, *lasR*, and *rhlR* (Figure S7A), and is suppressed by *daf-2(e1370)* but not *daf-16(mu86)* (Figure S7B). Thus, activation of *daf-2* signaling during *P. aeruginosa* infection results in the simultaneous downregulation of stress response and immune effectors genes that together could aid in the pathogenesis of these bacteria.

### *P. aeruginosa* infection causes delocalization of nuclear DAF-16

**Figure 4:**
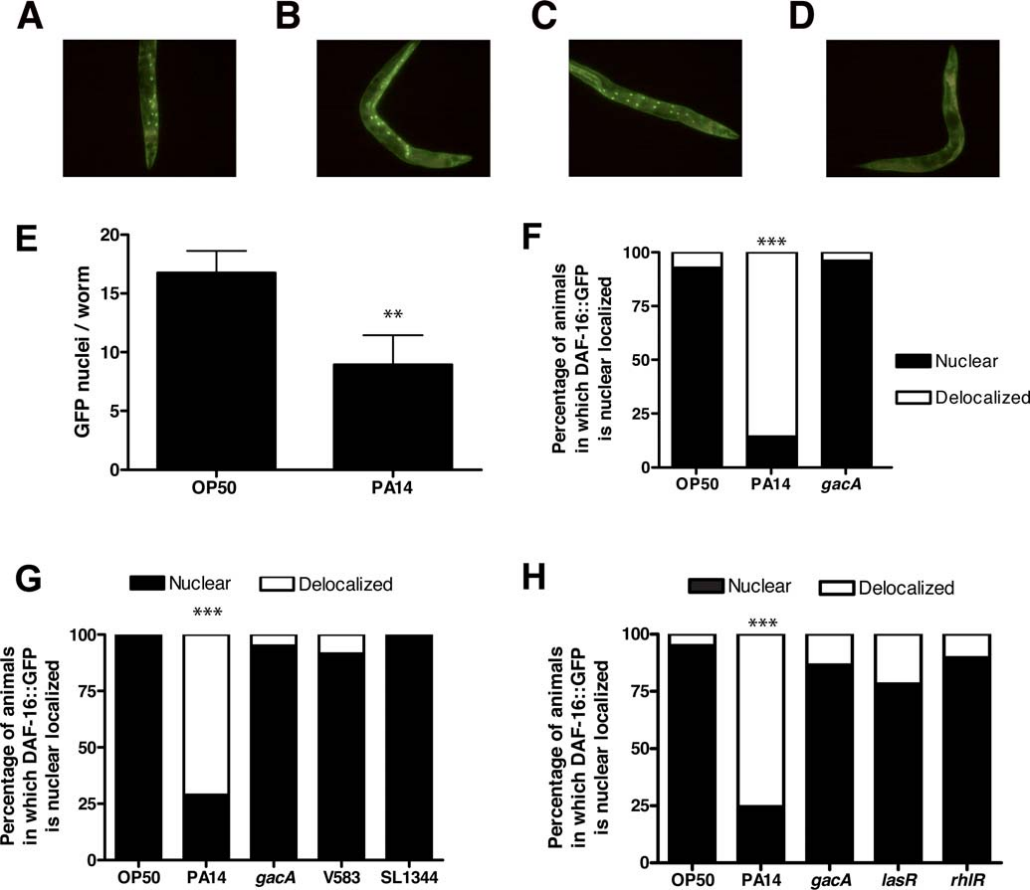
*P. aeruginosa* infection causes delocalization of nuclear DAF-16 in the intestine. (A–D) Representative fluorescence micrographs of DAF-16::GFP expressing adult worms with nuclear localized DAF-16::GFP fusion protein after exposure to (A) OP50-1 (B) PA14 *gacA*, and (C) wildtype PA14, and also (D) nuclear delocalized DAF-16::GFP in worms after exposure to wildtype PA14. (E) Mean number of nuclei with localized DAF-16::GFP after 18 hr exposure to either OP50-1 or PA14 following acute heat shock. ** t-test, p<0.001 (F–H) Proportion of worms in a population categorized as either showing predominately nuclear localized (“nuclear”) or predominately “delocalized” intestinal DAF-16::GFP. Nuclear localization was induced by either heat shock (70–90 minutes at 37°C) (F) or loss of germline proliferation (G–H) and then worms were exposed to (F–H) OP50-1, PA14, PA14 *gacA*, (G) SL1344, V583, (H) PA14 *lasR*, or PA14 *rhlR*. *** Fisher's exact test, p<0.0001.

The transcriptional effects of *daf-2* mutants are largely dependent on DAF-16 [16]. Under normal growth conditions, the DAF-2 pathway is active and DAF-16 protein is distributed predominately in the cytoplasm of every tissue. Conditions that reduce signaling in the DAF-2 pathway, including heat stress, ablation of the germline, and loss of *daf-2* function, cause DAF-16 protein to be localized in the nucleus [68],[73]. DAF-16 translocates from the nucleus to the cytoplasm when DAF-2 signaling is increased [73]. We determined the effect of PA14 infection on DAF-16 localization using transgenic worms that express a functional DAF-16::GFP fusion protein [73]. We confirmed that exposure to PA14 does not cause increased DAF-16 nuclear localization [7],[8]. Our gene expression data suggested that PA14 infection activates DAF-2 signaling and could consequently result in delocalization of DAF-16 from the nucleus. To measure this effect, it was necessary to first localize DAF-16 to the nucleus and then compare the reversal of that localization between infected and uninfected animals. We used two independent means to drive DAF-16::GFP to the nucleus: brief heat shock and removal of the germline, both of which are reported to induce DAF-16 nuclear localization in a DAF-2-independent manner [74],[75]. Heat shock causes transient nuclear localization that reverses over time, whereas the degree of nuclear localization caused by loss of germline proliferation is relatively stable in young worms. For the heat-shock approach, worms exposed to 37°C dry heat for 70 to 90 minutes were shifted onto plates containing OP50-1, PA14, or PA14 *gacA*. After 16 hours at 25°C, almost all of the worms exposed to OP50-1 (Figure 4A) or PA14 *gacA* (Figure 4B) retained nuclear DAF-16::GFP. By contrast, in a majority of the PA14-infected population, DAF-16::GFP was delocalized from the nuclei of intestinal cells (Figure 4C–4D). We quantified this effect by counting the number of intestinal nuclei in which DAF-16::GFP fluorescence was apparent. As shown in Figure 4E, the number of nuclei with visible DAF-16::GFP nuclear localization was significantly reduced in PA14-infected worms compared to OP50-fed worms (p<0.001). Because the distribution of nuclei containing DAF-16::GFP per worm was distinctly bimodal (representative examples shown in Figures 4C and 4D), individual worms could be classified as having predominately nuclear-localized or predominately nuclear-delocalized (i.e., cytoplasmic) DAF-16::GFP in subsequent assays. Following 16 hours recovery from acute heat shock, approximately 80% of PA14-infected worms no longer retained the DAF-16::GFP fusion protein in the nucleus, whereas worms exposed to OP50-1 or PA14 *gacA* were not significantly affected (Figure 4F, p<0.0001). DAF-16::GFP remains nuclear localized in heat-shocked worms exposed to OP50-1 or PA14 *gacA* for 48 to 60 hours.

To confirm that the rapid delocalization of DAF-16 is a general physiological response to PA14 infection and not an artifact specific to the use of heat shock stimulus to nuclear localize DAF-16, we examined the effect of PA14 infection on nuclear localized DAF-16 using worms in which germline proliferation was eliminated. Loss of germline proliferation causes nuclear localization of DAF-16 in the intestinal cells of adult worms [68]. RNAi knockdown of *cdc-25.1* produces worms that lack germline proliferation [76] and localizes DAF-16::GFP to intestinal nuclei. Nuclear localization of DAF-16 due to loss of germline proliferation requires *kri-1* [77]. We confirmed that knockdown of *cdc-25.1* by RNAi affects DAF-16::GFP nuclear localization through its effect on germline proliferation by showing that nuclear localization of DAF-16::GFP could be suppressed when *kri-1* was knocked down by RNAi (Figure S8). In adult worms without proliferating germline, PA14 infection caused delocalization of nuclear DAF-16 in approximately 75% of worms after 16 hours (p<0.0001), whereas worms exposed to OP50-1 or PA14 *gacA* were not significantly affected (Figure 4G). Delocalization of DAF-16::GFP upon PA14 infection was not due to a generalized loss of nuclear localization because we failed to observe a loss of nuclear integrity. Nor was it due to a loss of the ability to retain transcription factors in the nucleus because other nuclear localized GFP constructs, including a translational fusion of GFP to the GATA transcription factor ELT-2, remained nuclear over the course of the experiment (data not shown). In addition, DAF-16 nuclear delocalization caused by PA14 infection was reversible by subsequent heat shock (data not shown), indicating that PA14 infection does not simply render DAF-16 incapable of nuclear translocation or retention. Notably, the nuclear delocalization of DAF-16 occurred early during PA14 infection and affected DAF-16 in intestinal nuclei. Thus, delocalization of DAF-16 occurred in the appropriate time and location to account for the transcriptional patterns observed during PA14 infection, providing an independent category of evidence for the model that PA14 infection activates the DAF-2 insulin-like signaling pathway.

DAF-16 target genes were downregulated following infection by *P. aeruginosa* (PA14), but not by *E. faecalis* (V583) or *S. typhimurium* (SL1344) (Figure 1A). We therefore hypothesized that infection with V583 and SL1344 would not result in the translocation of DAF-16 from the nucleus. Because survival of worms on V583 and SL1344 is extended compared to PA14, delocalization of DAF-16 might occur later in worms exposed to V583 or SL1344 than in worms exposed to PA14. We therefore used worms lacking a proliferating germline to cause DAF-16 nuclear localization to ensure that nuclear localization of DAF-16 is distinguishable for at least 96 hours. Consistent with the failure of V583 and SL1344 to downregulate DAF-16 target genes (Figure 1A), there was no significant decrease in DAF-16 nuclear localization in worms exposed to these pathogens (Figure 4G). Finally, consistent with the requirement for *gacA*, *lasR*, and *rhlR* for the downregulation of immune gene expression during PA14 infection (Figure 1C), similar to the PA14 *gacA* mutant, the *rhlR* and *lasR* mutants also failed to cause significant delocalization of DAF-16::GFP compared to uninfected controls (Figure 4H). Thus, we can conclude that activation of DAF-2 affects the nuclear localization of DAF-16 and the transcription of DAF-2-dependent immune genes, such as *thn-2, lys-7, and spp-1*. This effect is specific to infection by *P. aeruginosa* and requires GacA-, LasR-, and RhlR-regulated virulence factors.

### DAF-2 and a DAF-2 agonist, INS-7, are required for *P. aeruginosa* to affect DAF-16 nuclear localization

**Figure 5:**
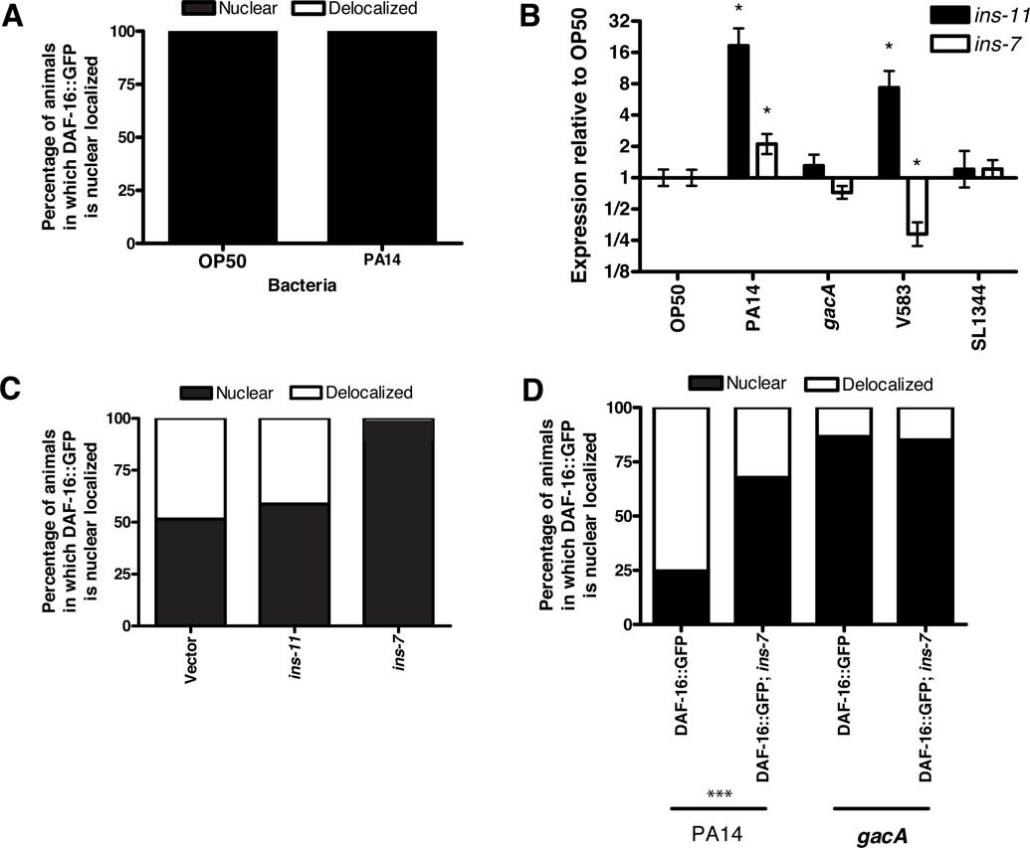
Insulin-like signaling is required for the delocalization of nuclear DAF-16 during *P. aeruginosa* infection. (A) Proportion of *daf-2(e1370)*; DAF-16::GFP worms exposed to OP50-1 or PA14 categorized as showing either predominately nuclear localized (“nuclear”) or predominately “delocalized” DAF-16::GFP. No nuclear delocalization is observed in *daf-2(e1370)* mutants. (B) Expression of insulin-like genes *ins-11* and *ins-7* in wildtype worms exposed to OP50-1, PA14, PA14 *gacA*, V583, and SL1344. (C) Proportion of worms in a population categorized as either showing predominately nuclear localized (“nuclear”) or predominately “delocalized” DAF-16::GFP. RNAi knockdown of *ins-11*, *ins-7* or vector control RNAi shown. (D) Proportion of DAF-16::GFP and DAF-16::GFP;*ins-7(tm1907)* worms in a population categorized as either showing predominately nuclear localized (“nuclear”) or predominately “delocalized” DAF-16::GFP. *** Fisher's exact test, p<0.0001.

The results presented thus far indicate that PA14 infection activates DAF-2, resulting in the translocation of DAF-16 from the nucleus and the DAF-2-dependent repression of immune gene expression. They further suggest that loss-of-function mutations in *daf-2* would suppress the delocalization of DAF-16. Indeed, when we compared DAF-16::GFP localization in *daf-2(e1370)*; DAF-16::GFP animals that were exposed to either OP50-1 or PA14, no significant difference in DAF-16::GFP localization was observed between PA14-infected and uninfected controls over the course of 4 days (Figure 5A). This indicates that *daf-2* is required for the delocalization of DAF-16 during PA14 infection and is consistent with the failure of PA14 to downregulate host effector genes in *daf-2(e1370)*. It further suggests that PA14 infection suppresses *C. elegans* immune gene expression by affecting host components that are upstream of *daf-2*.

*daf-2* encodes the only homolog of a mammalian insulin/IGF-1-family receptor in the *C. elegans* genome [78], and its activity is affected by insulin-like molecules [79],[80]. Therefore, insulin-like peptides are attractive candidates to be subverted by *P. aeruginosa* to activate DAF-2. As shown in Figure 5B, two insulin-like peptides, *ins-7* and *ins-11*, were upregulated in worms exposed to PA14. Several lines of evidence implicate *ins-7*—and not *ins-11*—as contributing to the activation of DAF-2 during PA14 infection. First, *ins-7* expression is not induced by exposure to either *E. faecalis* or *S. typhimurium*, but *ins-11* expression is induced by exposure to *E. faecalis* (Figure 5B). Also, *ins-7* is not induced in worms exposed to PA14 *gacA*, *lasR*, or *rhlR* mutants (Figure 5B and unpublished data). Moreover, *ins-7* is known to be an insulin agonist [16],[80]. Thus, the induction of *ins-7*, but not *ins-11*, is concordant with the activation of DAF-2 by PA14 but not *E. faecalis* infection. Second, the *ins-7* deletion mutant *ins-7(tm1907)* is resistant to PA14 infection, but the *ins-11* deletion mutant *ins-11(tm1053)* is not distinguishable from wildtype worms with respect to susceptibility to PA14 (Figure S9, Table S3). Lastly, RNAi knockdown of *ins-7* suppresses the effect of PA14 infection on DAF-16 nuclear delocalization, whereas *ins-11* RNAi knockdown has no distinguishable effect (Figure 5C). To confirm that *ins-7* is required for the effect of PA14 on DAF-16 localization, we examined DAF-16::GFP localization in the deletion mutant *ins-7(tm1907)*. Loss of *ins-7* suppressed the effect of PA14 infection on DAF-16 nuclear delocalization in worms lacking germline proliferation (Figure 5D), indicating that *ins-7* is required for the effect of PA14 on DAF-16 nuclear localization.

### DAF-16 is required in the intestine for defense against PA14

**Figure 6:**
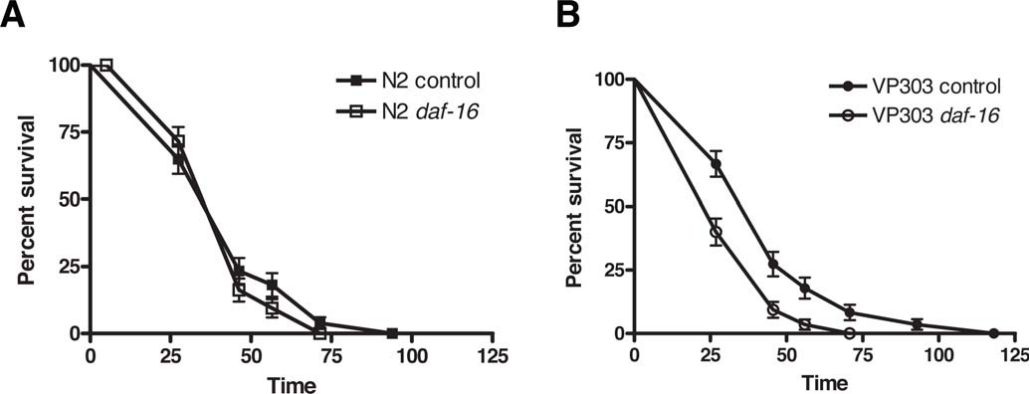
Intestinal DAF-16 is specifically required for resistance to *P. aeruginosa*. (A–B) Survival of worms cultured on PA14 lawns was monitored over time at 25°C. (A) In wildtype N2 worms, the effects on survival of RNAi knockdown of *daf-16* or control RNAi were indistinguishable (logrank, p = 0.85). (B) In VP303 worms, intestinally restricted RNAi knockdown of *daf-16* caused enhanced sensitivity to PA14 compared to control RNAi (logrank, p = 0.0002).

*daf-16* null mutants are indistinguishable from wildtype in their ability to survive infection by a variety of pathogens [8],[15],[23],[81]. However, the phenotype that results from the loss of gene function in an entire organism is the combination of the effects on various tissues; and the tissue required for *daf-16* in immune function has not been investigated. The profound effects of PA14 infection on insulin-like signaling, and the central role for DAF-16 in that interaction, led us to reevaluate the role of DAF-16 in defense against bacterial pathogens. Several lines of evidence suggested an important role for DAF-16 in the intestine. First, the intestine is the site of PA14 infection [82], and the major site of expression of host defense genes including *spp-1* and *lys-7* [7],[39]. Second, resistance to PA14 is associated with nuclear localization of DAF-16 specifically in the intestine. Loss of germline proliferation, which causes DAF-16-dependent resistance to PA14 (unpublished data), causes DAF-16 nuclear localization predominately in the intestine [68]. A forward genetic screen for enhanced resistance to PA14 identified mutants with enhanced nuclear localization of DAF-16 in the intestine [81]. Similarly, we have observed that loss of *ins-7* caused DAF-16::GFP nuclear localization primarily in intestinal cells (unpublished data). Third, DAF-16 nuclear delocalization during PA14 infection is most evident in intestinal cells. Thus, we sought to examine the function of intestinal DAF-16 by knocking down the expression of *daf-16* only in the intestine. Tissue-specific knockdown of gene expression can be achieved in *C. elegans* using strains in which the RNAi-deficient *rde-1* mutant is rescued by expressing a transgene carrying the *rde-1* gene in a specific tissue, such as intestine [83], hypodermis or muscle [84]. The strain VP303 expresses *rde-1* in the intestine of an *rde-1(ne219)* mutant background, allowing for intestine-restricted RNAi knockdown [83],[85]. VP303 and N2 worms are indistinguishable for resistance to PA14 (Table S4). As expected from previous reports, [8],[15],[23],[81], RNAi knockdown of *daf-16* in wildtype N2 worms had no effect on susceptibility to PA14 (Figure 6A). RNAi knockdown of *daf-16* in VP303 worms, however, caused enhanced susceptibility to PA14 (Figure 6B). To confirm the specificity of VP303 for intestine-specific knockdown, we exposed N2 and VP303 strains to bacteria that express double-stranded RNA corresponding to a muscle-specific gene *unc-22*, which encodes for twitchin that functions in the muscles to regulate the actomyosin contraction-relaxation cycle and to maintain normal muscle morphology [86]. As expected, RNAi against *unc-22* resulted in the canonical twitching phenotype in N2 but not in the VP303 strain. *unc-22* RNAi also has no effect on pathogen resistance in both the N2 and VP303 strains (Table S4). Recently, the hypodermal tissue has also been shown to contribute to immune response to a fungal pathogen [87]. Using the NR222 strain, in which RNAi knockdown is restricted to the hypodermis, we showed that loss of *daf-16* in the hypodermis is not required for pathogen resistance (Table S4). These results indicate that DAF-16 is required in the intestine for resistance to PA14, whereas loss of DAF-16 in the intestine, in combination with loss of DAF-16 in non-intestinal tissues, has an overall neutral effect on the ability of worms to defend against PA14 infection. Intestinally but not hypodermally expressed DAF-16 is essential for resistance to PA14, a function that was previously masked in studies examining the requirement for DAF-16 in the whole worm.

## Discussion

### *Pseudomonas* infection suppresses host defense genes

Models of host-pathogen interactions predict that pathogen challenge will result in the induction of host defense genes. Repression of host defense genes is often associated with suppression of host defense pathways by the pathogen. Using quantitative RT-PCR, we confirmed that known host defense effectors, including *thn-2*, *lys-7*, and *spp-1* are downregulated during PA14 infection (Figure 1A). Because knockdown of basal expression of *thn-2, lys-7, and spp-1* by RNAi resulted in enhanced susceptibility to PA14 (Figure S1), suppression of their expression during infection should compromise host defense. The observation that knockdown of *thn-2*, *lys-7*, and *spp-1* by RNAi causes enhanced sensitivity to PA14 despite the downregulation of their expression in PA14-infected worms is explained in part by the fact that expression levels drop following infection whereas expression levels are already reduced to low levels when RNAi-treated worms are exposed to PA14. The downregulation of these host defense effectors is not a typical response of *C. elegans* to pathogen challenge; the expression of these host effectors was induced or unaffected in response to *E. faecalis* and *S. typhimurium* (Figure 1A). A large number of host defense effectors are efficiently induced in response to PA14 infection, suggesting that the downregulation of a particular set of host defense genes is a specific effect (Figure 1B and Table S2). Also, repression of a specific subset of host defense effectors by PA14 is likely to represent a specific interaction between *P. aeruginosa* and *C. elegans* because PA14 requires the virulence regulatory genes *gacA*, *lasR* and *rhlR* to suppress host defense genes (Figure 1C). *gacA*, *lasR* and *rhlR* mutants are each attenuated in virulence (Table S1), suggesting a link between virulence and the downregulation of host defense effectors. The specific requirement of GacA-, LasR- and RhlR-regulated virulence factors in immune suppression is supported by the observation that the PA14_23420, PA14_23430, PA14_59010 mutants that are attenuated in virulence are not defective in immune suppression (Figure S3B). As an initial effort to define the GacA-, LasR- and RhlR-regulated virulence factors that are required for immune suppression, we showed that *dsbA, pqsA* and the T3SS, all of which have been shown to be under the control of the GacS/GacA two component regulator and the quorum-sensing system [49], [50], [52]–[54] are not required for the downregulation of *lys-7* expression (Figure S3B). The hypothetical protein, PA14_41070 that contains the S-adenosyl-methionine (SAM)-dependent methyltransferase domain that is required for downregulation of *Drosophila* NF-κB-regulated antimicrobial peptides expression [36] is also dispensable for immune suppression in *C. elegans* (Figure S3B). Overall these results suggest that the ability of *P. aeruginosa* to suppress *C. elegans* host defense effectors requires a subset of GacA-, LasR- and RhlR-regulated virulence factors and are not merely reflections of attenuation of virulence.

We have thus provided evidence that we have identified a new mechanism of host immune suppression. This mechanism does not require the SAM-dependent methyltransfrease or the type III secretion system, which have been previously implicated in host immune suppression through their effects on NF-κB and MAPK signaling [36],[43],[44]. Instead, it requires the GacA two-component regulator and the LasR and RhlR quorum-sensing regulators to regulate factors necessary to subvert host defense. These factors, which we showed are likely to be independent of DsbA and quinolone signaling, could potentially be identified by screening for *P. aeruginosa* mutants that fail to downregulate the expression of host defense effectors, a strategy that we are actively pursuing. Finally, as discussed below, this immune suppression is mediated by the host insulin-like signaling pathway, instead of the MAPK and NF-κB pathways.

### *Pseudomonas* infection activates insulin-like signaling

**Figure 7:**
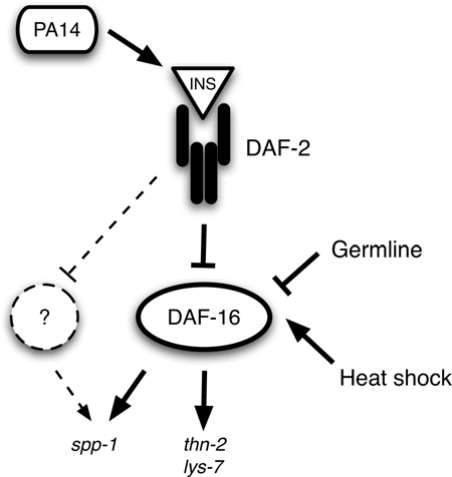
Model of *P. aeruginosa* activation of insulin-like signaling. PA14 infection causes an insulin agonist-mediated activation of DAF-2, which likely involves expression of *ins-7*. Upon DAF-2 activation, several serine-threonine kinases phorphorylate DAF-16, causing the transcription factor to be retained in the cytoplasm and inhibiting its transcriptional-regulatory activity. Consequently, DAF-2–regulated gene expression is affected, causing the downregulation of immune defense genes such as *lys-7* and *thn-2*. Germline signaling and heat shock regulate DAF-16 in parallel to the effect of PA14 and DAF-2 activity. DAF-2 and DAF-16 may function in a network with other signaling components to affect the expression of DAF-2-regulated genes including *spp-1*.

The specific downregulation of the host defense effectors *thn-2, lys-7, and spp-1* during *P. aeruginosa* infection (Figure 1A) suggested that a host defense signaling pathway was being subverted by *P. aeruginosa* to repress host defenses. Gene expression and host protein localization studies support a model that PA14 infection activates the DAF-2/DAF-16 insulin-like signaling pathway, resulting in the downregulation of DAF-16-regulated immune genes (Figure 7). The downregulation of *thn-2, lys-7, and spp-1* during PA14 infection was abolished in *daf-2* loss-of-function mutants (Figure 3A–3C). The requirement for *daf-2* for gene downregulation during infection held true when the gene expression analysis was extended to include the entire genome by microarray (Figure 2A) or to a set of 146 candidate immune and stress genes that were measured more precisely by qRT-PCR (Figure 2C). Comparison of the transcriptional response to infection in mutants of each of the p38 MAPK, Sma/TGF-β, and insulin-like signaling pathways to the response in wildtype N2 worms confirms that the insulin-like signaling mutant, *daf-2(e1370)*, suppresses a portion of the wildtype response to PA14 infection in a fashion distinct from the effects of loss of p38 MAPK or TGF-β signaling (Figure 2C–2E).

The model we propose in Figure 7 predicts that during PA14 infection, activation of insulin-like signaling will result in the inhibition of DAF-16. Inhibition of DAF-16 by insulin-like signaling is mediated by phosophorylation of DAF-16 by serine threonine kinases that are homologous to mammalian AKT and SGK, and results in the cytoplasmic retention of DAF-16 [68],[73]. We used several assay conditions to examine the nuclear localization of DAF-16 during PA14 infection, providing evidence that PA14 infection causes DAF-16 nuclear delocalization (Figure 4). Based on the transcriptional response data (Figure 1), we further predicted that nuclear delocalization would occur in response to wildtype PA14, but would not occur in response to infection by *E. faecalis* or *S. typhimurium*, and that PA14 mutants of *gacA*, *lasR* and *rhlR* would also be defective for the ability to cause delocalization of nuclear DAF-16. Each of these predictions was confirmed when tested (Figure 4G–4H), suggesting that DAF-16 delocalization is a PA14-infection specific effect. The model also predicted that while stimuli that function in parallel to insulin-like signaling to cause DAF-16 nuclear localization can be reversed by PA14 infection, such as heat shock and loss of germline proliferation, mutations that disrupt insulin-like signaling would also disrupt the DAF-16 nuclear delocalization caused by PA14. We confirmed that nuclear localization of DAF-16 in the insulin-like receptor mutant *daf-2(e1370)* was not affected by PA14 infection (Figure 5A). Finally, we showed that PA14 infection, but not infection by *E. faecalis*, *S. typhimurium*, or PA14 *gacA* caused increased expression of the insulin-like peptide *ins-7*, suggesting that *ins-7* may participate in signaling upstream of *daf-2* in response to PA14 infection (Figure 5B). Consistent with this hypothesis, we found that a deletion mutant of *ins-7* attenuated the ability of PA14 to cause nuclear delocalization of DAF-16 (Figure 5C). Collectively, the expression profile and DAF-16 localization data indicate that PA14 infection induces the expression of *ins-7* to activate insulin signaling that leads to inhibition of DAF-16 and downregulation of immune gene expression.

In a separate report, we showed that a neuroendrocine signaling axis that functions upstream of the DAF-2/DAF-16 pathway to regulate lifespan [88],[89] and dauer formation [90] also regulates innate immunity. Regulation of innate immunity is mediated by the expression of *ins-7* in the neurons (unpublished data). We showed that mutants with decreased neurosecretion, such as *unc-64(e246)* and *unc-31(e928)*, are more resistant to PA14 because they express significantly higher levels of immune effectors due to constitutive nuclear localization of intestinal DAF-16. In that report, we tested whether neuronal function was required for activation of DAF-2 by PA14 infection. We found that constitutive nuclear localization of DAF-16::GFP in neurosecretion defective mutants, such as *unc-64(e246)* is not reversed by exposure to PA14 (data not shown). Taken together, the data indicate neuronal secretion of an insulin-like peptide, INS-7, contributes to activation of DAF-2 insulin-like signaling during PA14 infection. It will be interesting to determine how PA14 infection induces the expression of *ins-7* in neurons and thereby suppress immune gene expression.

### Other contributors to the transcriptional response to *Pseudomonas*

Activation of DAF-2 appears to account for a substantial proportion of the downregulation of host defense genes during PA14 infection, but is less important for the upregulation of host defense genes (Figure 2A and 2C). To understand the mechanisms contributing to gene upregulation during infection, we also examined the contributions of the Sma/TGF-β and p38 MAPK pathways to the transcriptional response to PA14 (Figure 2B,2D,2E). For many genes, the transcriptional response to PA14 is intact in mutants of all three pathways tested (Table S2). This indicates that the regulation of the transcriptional response to PA14 is redundant and/or that additional pathways contribute substantially to the transcriptional response to PA14. Functional redundancy in the regulation of host immune effectors is a conserved feature of innate immunity [91]. In light of this observation, it is especially notable that the downregulation of a substantial number of genes is dependent on DAF-2 insulin-like signaling.

The suppression of *spp-1* by PA14 (Figure 3C), unlike the suppression of *thn-2* and *lys-7* (Figure 3A and 3B), is not entirely DAF-16 dependent, suggesting additional layers of complexity in the regulation of DAF-2 target genes. Neither p38 MAPK nor Sma/TGF-β is required for the downregulation of *spp-1* (Figure S4 and Table S2). The factor that contributes to the downregulation of *spp-1* remains unknown, but may function downstream of DAF-2 in cooperation with DAF-16 (Figure 7).

A number of transcriptional co-regulators have been identified which regulate lifespan and stress resistance in conjunction with DAF-16 [75],[92],[93]. We considered two transcription factors, ELT-2 and SKN-1, as likely candidates because they are also expressed in the intestine. *elt-2* encodes a GATA transcription factor that is required for a substantial portion of the transcriptional response to PA14 [7], and is required to protect *C. elegans* from infection by pathogens [7],[23]. However, the pathways that act upstream of ELT-2 in the regulation of immune defense has not be elucidated. Intriguingly, a CTTATCA (reverse complement: TGATAAG) DNA motif that is enriched in the 5-prime flanking regions (5′-flank) of DAF-2/DAF-16-regulated genes [16] is essentially identical to a GATA-like motif (TGATAAGA) that is enriched in the 5′-flank of genes that are significantly up- or down-regulated in worms exposed to *P. aeruginosa* [7], and each is a derivative of the consensus GATA motif (WGATAR). For example, the 5′-flank of *spp-1*, *thn-2*, *gst-4*, *lys-7*, and *sod-3* each contain GATA motifs. This motif is distinct from the canonical FOXO binding site (TRTTTAG), which has been shown to bind DAF-16 *in vitro* [70]. The FOXO-binding DNA motif is present in the 5′-flank of *lys-7* and *sod-3*, but not *spp-1*, *thn-2*, or *gst-4*. Moreover, knockdown of *elt-2* by RNAi reduces the expression of *lys-7* and *spp-1* under uninfected conditions (data not shown) and enhances the relative downregulation of *thn-2* during PA14 infection [7]. Overall, these findings suggest that ELT-2 or another GATA-motif-binding transcription factor may function downstream of DAF-2 together with DAF-16 to regulate the expression of immune-response gene. One instantiation of the consensus-binding motif of the transcription factor SKN-1 (ATGATAAT) is remarkably similar to the WGATAR motifs found upstream of genes regulated by DAF-2 and PA14 infection. Recently, it has recently been shown that for lifespan SKN-1 is regulated by insulin-like signaling [93]. We observed that knockdown of *skn-1* by RNAi throughout the life of the animal caused enhanced sensitivity to PA14 (unpublished data). Thus, we wondered whether SKN-1 might contribute to DAF-2-dependent regulation of gene expression following PA14 infection. Knockdown of *skn-1* by RNAi does not affect the downregulation of *thn-2*, *spp-1*, or *lys-7* (data not shown). *gst-4* is known to be SKN-1-regulated. However, like *thn-2*, *spp-1*, and *lys-7*, knockdown of *skn-1* expression by RNAi does not abolish the downregulation of *gst-4* following PA14 infection, despite causing a reduction in the basal level of *gst-4* expression (Figure S7C). Future work must focus on determining transcription factors in addition to DAF-16 that accounts for the DAF-2-dependent downregulation of host defense genes.

We also note that not every downregulated gene is dependent on DAF-2 insulin-like signaling. For example, following PA14 infection, *acdh-1* is strongly repressed in wildtype and all of the mutants tested, including *daf-2(e1370)* (Table S2). This suggests that factors in addition to DAF-2 insulin-like, Sma/TGF-β, and p38 MAPK signaling may contribute to the repression of host defense genes during PA14 infection. However, *acdh-1* expression is also downregulated in worms exposed to *E. faecalis, E. carotovora, P. luminescens, S. marcescens, S. typhimurium* and PA14 *gacA* (M. Nandakumar and M.-W. Tan, unpublished data, and [21]), suggesting that the downregulation of *acdh-1* may represent a different phenomena than the repression of other host defense genes.

### Insulin-like signaling regulates intestinal immune defense

Given the dynamic regulation of insulin-like signaling during infection, and particularly the complex role for DAF-16 in this interaction, we wondered why null mutants of *daf-16* are nonetheless indistinguishable from wildtype animals for their ability to survive infection by a variety of bacterial pathogens [8],[15],[23],[81]. Expression of *daf-16* in the intestine partially enhanced pathogen resistance of a *daf-16(mu86)*;*daf-2(e1370)* worm (unpublished data), consistent with the intestine being the site of infection, and the major site of immune gene expression. Additional observations that germline signaling, neuroendocrine signaling, and PA14 infection primarily modulate DAF-16 nuclear localization in the intestine highlighted the importance of intestinal DAF-16. A compensatory mechanism or other complex interaction acting across tissues could potentially mask the function of *daf-16* in the intestine. Using of intestine-specific *daf-16* knockdown by RNAi we show that DAF-16 is required for a protective response against pathogen. Supporting this finding further is the observation that enhanced resistance of *ins-7* to PA14 is associated with constitutive nuclear localization of DAF-16 specifically in the intestine (unpublished data).

The most parsimonious explanation for the failure to observe enhanced sensitivity to pathogens when *daf-16* is knocked down or knocked out in the whole organism is that intestinal and non-intestinal DAF-16 have antagonistic effects on host defense. Given the known complex regulation of DAF-16, this scenario is plausible. Balanced antagonistic effects on DAF-16 are observed in the context of signaling from the *C. elegans* germline and somatic gonad that regulate aging. Signals from the germline that normally suppress DAF-16 activity are balanced by distinct signals from the somatic gonad that normally enhance DAF-16 activity. The gonad signal appears to operate via the neurons and DAF-2 insulin-like signaling, while the germline signal appears to operate through a parallel pathway that does not require DAF-2 [94]. In this context, it is most appropriate to describe the effects of genetic manipulations in terms of net aggregate effects. Thus, the net aggregate effect of loss of *daf-16* in the entire organism is neutral while the net aggregate effect of loss of *daf-16* in the intestine results in pathogen sensitivity.

Endodermal GATA transcription factors have been shown to have a conserved role in regulating epithelial innate immune responses in the *C. elegans* intestine and human lung epithelial cells [7]. Here we find that *P. aeruginosa* can suppress epithelial immunity by affecting DAF-16 activity in the intestinal cells. The regulation of FOXO/DAF-16 by insulin-like signaling is highly conserved across organisms (reviewed in [95]). Given the medical importance of *P. aeruginosa*, for example in cystic fibrosis patients where it chronically infects lung epithelial cells [25],[26], it will be interesting in the future to investigate whether insulin-like signaling regulates an epithelial immune response and whether *P. aeruginosa* suppresses this immune response in human patients.

## Materials and Methods

### Worm and bacterial strains

*Caenorhabditis elegans* and bacterial strains are described in Table S5. Double mutants were constructed and verified using standard genetic and molecular methods [96]. Bacteria expressing dsRNA directed against *daf-16*, *lys-7*, *thn-2*, and *spp-1* was part of a *C. elegans* RNAi library expressed in *E. coli* (Geneservice, Cambridge, U.K.). Bacteria expressing dsRNA directed against *sod-3*, *ins-7*, *ins-11*, *kri-1*, and *skn-1* were part of a *C. elegans* RNAi library expressed in *E. coli* (Open Biosystems, Huntsville, Alabama). All bacterial strains were cultured under standard conditions.

### Microarray analysis

Growing worms for microarrays, RNA extraction, microarray hybridization, and data processing were performed as previously described [7]. Reference RNA and microarray hardware were matched to allow direct comparison of transcriptional response to PA14 in all worm strains. In particular, *sma-6(wk7)* experiments were performed in parallel with previously reported N2 experiments [7]. *daf-2(e1370)* animals were grown at 20°C until young adulthood and then placed for 24 hr at 25°C on lawns of either OP50-1 or PA14 grown on modified NGM. Microarray production, hybridization, and scanning were performed at the Stanford Functional Genomics Facility. cDNA was generated from total RNA. Experimental cDNA was labeled with Cy3 and reference cDNA was labeled with Cy5. Four replicates of each condition were examined. Microarray data was deposited in the Stanford Microarray Database [97]. The default background subtraction and normalization settings were used. Spots were filtered based on foreground to background ratio; values less than 1.2 were flagged. Log (base 2) values were exported. Gene expression on OP50-1 and PA14 were compared by t-test. Differences of 2 fold with p-values<0.05 were considered significant. Sensitivity analysis was conducted with a range of significance levels and fold-difference values to confirm that the choice of thresholds did not affect our conclusions.

### Quantitative RT-PCR

Age-matched young adult worms were exposed to bacterial lawns of OP50-1 or PA14 on NGM for 12 hours. RNA extraction and quantitative RT-PCR (qRT-PCR) was performed as previously described [7]. Briefly, 25 µl reactions were performed using the iScript One-Step RT-PCR kit with SYBR green according to the manufacturer's instructions (BioRad Laboratories, Hercules, CA), primers at a final concentration of 1 µM, and a data acquisition temperature of 76°C. In order to control for variation in RNA loading concentration, cycle threshold (Ct) values were normalized to three primer pairs (*ama-1*, F44B9.5, and pan-actin (*act-1,3,4*)) that were found to not change with infection. Summary statistics and statistical tests were calculated from N2-normalized cycle threshold values prior to conversion to relative fold change. Calculations were performed with a custom Perl script, Excel and R.

A panel of 146 infection and stress response genes queried by qRT-PCR included representatives of the antibacterial factor related (*abf*), lysozymes (*lys*), saposin-like proteins (*spp*), or thaumatin family (*thn*) gene classes, as well as genes identified to be transcriptionally regulated by PA14 [7] or by the DAF-2/DAF-16 pathway [16]. Whenever possible, primers were designed to amplify a sequence found in spliced cDNA but not genomic DNA by having one of the primer pairs overlap an exon junction. To this end, primer design was aided with the program AutoPrime [98]. Primer sequences are available from the authors upon request.

### Analysis of induction and repression responses to PA14 infection

To compare the response to infection in wildtype and mutant worms, RNA concentrations were measured in matched samples of worms exposed to OP50-1 and PA14. The RNA concentration of gene *g* in worms exposed to OP50-1 (designated OP50*_g_*) indicates the basal level of expression. The RNA concentration of gene *g* in worms exposed to PA14 (designated PA14*_g_*) indicates the level of expression that follows infection. The response to infection for gene *g* is the difference in RNA levels between OP50-1 and PA14 samples. This was quantified as *x_g_* = PA14*_g_* – OP50_g_ for qRT-PCR cycle threshold measurements and log_2_-converted microarray measurements. These log_2_-scale values were interpreted as fold changes using the conversion fold change = 2*^x^*. Positive values of *x_g_* (PA14*_g_* – OP50*_g_*>0) indicate induction of the expression of *g* in response to infection. Negative values of *x_g_* (PA14*_g_* – OP50*_g_*<0) indicate repression of the expression of *g* in response to infection.

An ordered set of *x_g_* values for a set of genes (such as the set of induced or the set of repressed genes) from a particular worm strain forms a vector designated X_strain_. Correlation analysis can be used to determine the effect of a mutation on the induction or repression response to PA14 infection. The squared correlation between X_wildtype_ and X_mutant_, r^2^, indicates the degree to which induction or repression is conserved in the mutant strain. Values of r^2^ that are statistically indistinguishable from 0 indicate that induction or repression is strongly attenuated.

For a more sensitive analysis, X_wildtype_ and X_mutant_ were compared by paired t-test. The summary statistic X_Δ_ was calculated as the average of X_mutant_ – X_wildtype_. X_Δ_ indicates both the magnitude and direction of the difference in induction or repression in response to infection between wildtype and mutant strains. For induced genes, X_Δ_<0 indicates attenuation of induction. For repressed genes, X_Δ_>0 indicates attenuation of repression. The significance of X_Δ_ values was determined by paired t-test.

### DAF-16 nuclear localization assay

Acute heat shock was performed at 37°C for 70 to 90 minutes. Precise exposure times were determined empirically during each run by qualitative assessment of worms after heat shock. Germline proliferation was disrupted by RNAi knockdown of *cdc-25.1* as previously described [76]. Worms were then exposed to PA14 or controls for approximately 16 hours; times varied from 14 to 18 hours as needed. Individual worms were classified as exhibiting predominantly nuclear DAF-16::GFP or predominantly delocalization DAF-16::GFP under 160× total magnification using an Olympus SZX12 dissecting microscope with a 1.6× objective and an EN GFP-LP filter cube (catalog no. 41018, Chroma Technology, Brattleboro, VT) which has the following specification: excitation band-pass filter 470±20 nm, emission filters >495 nm long pass followed by another >500 nm long pass. Fluorescence micrographs were collected at 200× magnification by fluorescence microscopy using a Leica DMRXA2 microscope with the Leica I3 filter set (Excitation 420 nm, Emission 525 nm, 30 nm band pass) for GFP. The criteria for predominately nuclear was the greater than 4 intestinal nuclei with nuclear localized DAF-16::GFP. The proportion of worms in each category is a metric for comparing the extent of nuclear localization of DAF-16 between populations, and have previously been used to examine the genetic control of DAF-16 nuclear localization in other contexts [99]. Assays scored in this fashion are highly reproducible between experiments and across experimenters.

### *C. elegans* survival assays

Assays to determine the ability of *C. elegans* to survive PA14 infection were performed as described [76]. Briefly, to avoid the confounding effects of progeny production and internal hatching on survival, sterile worms were used in survival assays. To sterilize worms, *rrf-3(pk1426);glp-4(bn2)* and *pha-1(e2123)* were raised at 25°C. *ins-7(tm1907)*, *ins-11(tm1053)*, and wildtype controls were sterilized using RNAi knockdown of *cdc-25.1*. VP303 and NR222 worms are resistant to RNAi in the germline. Thus, VP303, NR222 and N2 worms were grown at 20°C and shifted to 27.5°C for 24 hours at the L3/L4 molt, which causes sterility. PA14 was grown overnight in King's Broth containing 100 mg/ml rifampicin at 37°C. 10 µl was spread on modified NGM and grown for 24 hours at 37°C. Worms were infected at 25°C by feeding on PA14 lawns. Kaplan-Meier survival analysis was performed using StatView 5.0.1. The Mantel-Cox logrank test was used to assess statistical significance of differences in survival. Only p-values<0.01 were considered significant. Mean time to death and standard error of the mean was calculated in StatView and then normalized to N2 for graphical comparison (Tables S1, S3, and S4).

## Supporting Information

Text S1 Supplemental Text
(13 KB PDF)

Figure S1 Knockdown of *thn-2*, *lys-7*, and *spp-1* by RNAi enhances the susceptibility of *C. elegans* to *P. aeruginosa* infection. Survival of worms in which (A) *spp-1*, (B) *lys-7*, or (C) *thn-2* was knocked down by RNAi followed by exposure to wildtype PA14 at 25°C was monitored over time. *rrf-3(pk1426);glp-4(bn2)* worms were used to enhance sensitivity to RNAi and to prevent progeny production, which can confound pathogen survival assays.
(417 KB TIF)

Figure S2 *P. aeruginosa gacA*, *lasR*, and *rhlR* are required immune suppression in *C. elegans* mutants that are unable to limit bacterial accumulation. Expression of downregulated host defense effectors genes in *tnt-3(aj3)* worms exposed to the PA14 and PA14 *gacA*, *lasR*, and *rhlR* mutants. Mean transcript levels are plotted relative to matched controls exposed to OP50-1. Error bars indicated SEM. At least 3 replicates of each condition were examined. * t-test, p<0.05 comparison to *tnt-3(aj3)* exposed to OP50-1.
(166 KB TIF)

Figure S3 Many *P. aeruginosa* virulence factors are not required for immune suppression in *C. elegans*. (A) Expression of the host defense effector *lys-7* and *spp-1* in wildtype worms exposed to wildtype PA14 and the PA14 mutant *pscD* and PA14_41070 measured by qRT-PCR. Mean transcript levels are plotted relative to matched controls exposed to OP50-1. Error bars indicated SEM. 3 replicates of each condition were examined. * t-test, p<0.05 (B) Expression of the host defense effector *lys-7* in wildtype worms exposed to wildtype PA14 and the PA14 mutants *gacA*, *lasR*, *rhlR*, *exoU*, *exoT*, *exoY*, *pscD*, PA14_41070, *dsbA*, *pqsA*, PA14_23420, PA14_23430, and PA14_59010 measured by visual classification of *lys-7::GFP* fluorescence as high or low intensity at 100× total magnification. Expression of *lys-7::GFP* in uninfected worms was the standard for high intensity fluorescence. Data is also shown in Table S1. * t-test, p<0.05 (C–D) Fluorescence micrographs of *lys-7::GFP* worms categorized as (C) high or (D) low expression.
(442 KB TIF)

Figure S4 2×2 factorial interaction plots depicting the contribution of p38 MAPK to the transcriptional response to *P. aeruginosa*. Both p38-dependent (A–F) and p38-independent (G–K) induction and repression of gene expression in response to PA14 infection are observed. Mean (and SEM) of log_2_ scale transcript levels relative to N2 OP50-1 were plotted for (A) *clec-85*, (B) *lys-1*, (C) *lys-8*, (D) F35E12.8, (E) Y40D12A.2, (F) *gst-38*, (G) *lys-2*, (H) *cpr-3*, (I) *spp-18*, (J) F55G11.2, (K) and T10D3.6 in N2 (blue) and *sek-1(km4)* (red) exposed to OP50-1 and PA14.
(525 KB TIF)

Figure S5 Knockdown of *thn-2*, *lys-7*, and *spp-1* by RNAi decreases the resistance of *daf-2(e1370)* mutants to colonization by P. aeruginosa. RNAi knockdown of *thn-2*, *lys-7*, and *spp-1* resulted in increased *P. aeruginosa* accumulation in *daf-2(e1370)* worms compared to control RNAi (Fisher's exact test; p = 0.0035, p = 0.0054, and p<0.0001, respectively). RNAi knockdown of *sod-3* did not significantly affect colonization (Fisher's exact test, p>0.9999). RNAi or control treated *daf-2(e1370)* were exposed to a PA14 strain that expresses GFP (PA14-GFP) as young adults for 108 hr. Individual worms were classified as having detectable or undetectable GFP fluorescence in the intestinal lumen by visual inspection at 200× total magnification. For each RNAi treatment, a total of 65 worms were assayed blind.
(193 KB TIF)

Figure S6 *sod-3* expression is repressed following *P. aeruginosa* infection. qRT-PCR measurement of *sod-3* transcript levels in N2 worms exposed to OP50-1 and PA14 is plotted relative to OP50-1 levels. * t-test, p<0.05.
(734 KB TIF)

Figure S7 The expression of *gst-4* is repressed in worms exposed to *P. aeruginosa* in a *skn-1*-independent manner. (A) Fluorescence intensity of *gst-4*::*GFP* in worms exposed to wildtype PA14 and the PA14 mutants *gacA*, *lasR* and *rhlR*. Worms of uniformly bright GFP intensity were placed on PA14 for 24 hours. Individual worms were scored for GFP intensity in three categories: low, medium, and high. Population proportions are significantly different in all pairwise comparisons with the exception of *lasR* and *rhlR*, which are statistically indistinguishable (Chi-square test, p<0.05). (B) Expression of *gst-4* measured in N2, *daf-2(e1370)*, *daf-16(mu86)*, and *daf-16(mu86)*;*daf-2(e1370)* exposed to OP50-1 and PA14. Mean transcript levels were plotted relative to N2 OP50-1. Error bars represent SEM. t-test ˆ p<0.05 comparison to N2 PA14, + p<0.05 comparing OP50-1 to PA14. (C) Mean (and SEM) of log_2_ scale transcript levels relative to N2 OP50-1 for *gst-4* in *skn-1* RNAi and vector control worms exposed to OP50-1 and PA14.
(437 KB TIF)

Figure S8 *kri-1* is required for nuclear localization in *cdc-25.1* RNAi-treated animals without proliferating germlines. The localization of DAF-16 in animals exposed first to *cdc-25.1* RNAi and then *kri-1* RNAi was assayed. *** Fisher's exact test, p<0.0001.
(721 KB TIF)

Figure S9 *ins-7* but not *ins-11* is required for resistance to *P. aeruginosa*. Survival of N2, *ins-7(tm1907)* and *ins-11(tm1053)* worms was monitored on PA14 over time at 25°C. *ins-7(tm1907)* is significantly more resistant to PA14 than N2 or *ins-11(tm1053)* (logrank, p<0.0001 and p = 0.0002, respectively). *ins-11(tm1053)* is statistically indistinguishable from N2 (logrank, p = 0.0902).
(164 KB TIF)

Figure S10 *P. aeruginosa* exposure negatively regulates host defense *daf-2* target genes. (A) Venn diagram representing the intersection of the “broad” *daf-2* and PA14 consensus datasets. Values are fold enrichment over chance. The greatest enrichment of genes that are affected in both *daf-2* mutants and during PA14 infection was found in the inversely regulated categories. (B) Venn diagram representing the intersections of the “broad” *daf-2* and PA14 datasets among immune genes. All immune genes that are affected in both *daf-2* mutants and during PA14 infection are found in the inversely regulated categories.
(236 KB TIF)

Table S1 Survival of *pha-1(e2123)* worms and *lys-7::GFP* expression in transgenic worms following exposure to PA14 and PA14 mutants.
(6 KB PDF)

Table S2 Relative magnitude of induction or repression following 12 hr exposure to PA14 (PA14 - OP50) in log base-2 scale.
(40 KB XLS)

Table S3 *ins-7*, but not *ins-11*, is required for resistance to PA14. Worms were sterilized by RNAi knockdown of *cdc-25.1*. Glp worms were exposed to PA14 and survival was monitored over time.
(5 KB PDF)

Table S4 Intestinal-specific knockdown of *daf-16* causes enhanced susceptibility to PA14. Worms were sterilized by 27.5°C treatment.
(6 KB PDF)

Table S5 Strains.
(20 KB PDF)

Table S6 Meta-analysis of DAF-2 pathway and PA14 microarray datasets.
(761 KB XLS)

Table S7 DAF-2-regulated genes are enriched for discordantly regulated infection-response genes.
(6 KB PDF)

Table S8 List of immune effector genes: genes encoding proteins with antimicrobial activity and/or required to protect *C. elegans* from *P. aeruginosa* infection.
(17 KB PDF)
