# Supplementary material for: Pseudomonas aeruginosa Suppresses Host Immunity by Activating the DAF-2 Insulin-Like Signaling Pathway in Caenorhabditis elegans
Source: PLoS Pathog. 2008 Oct 17;4(10):e1000175. doi: 10.1371/journal.ppat.1000175 (PMC2568960; doi:10.1371/journal.ppat.1000175)
Supplement: Text S1 — Supplemental Text (13 KB PDF) [file ppat.1000175.s001.pdf]

Supplemental Text

To confirm that genes regulated in common between PA14-infected and *daf-2* loss-of-function mutants tend to be regulated in opposite directions [1], we compared significantly up- and down-regulated genes from two independent *daf-2* pathway microarray datasets [2,3] to three PA14 infection microarray datasets [1,4]. Microarray gene sets were obtained from published supplemental data tables. Murphy *et al.* [3] and Shapira *et al.* [1] each used custom PCR microarray platforms, whereas McElwee *et al.* [2] and Troemel *et al.* [4] used the Affymetrix GeneChip technology. Gene names were corrected by comparison to Wormbase release 170. We created consensus datasets for *daf-2*- and PA14-regulated genes based on the union of each individual dataset, which we termed the “broad” set (Table S6). The combined PA14 datasets were created from the Shapira et al. and Troemel et al. PA14 data sets. The combined *daf-2* datasets were created from the Murphy et al. and McElwee et al. *daf-2* data sets. By comparing the direction of regulation of genes contained in each dataset, we can determine whether PA14 infection and loss of *daf-2* function concordantly or discordantly regulate a common set of genes (Figure S10A). Fold enrichment for gene set intersections was calculated as the ratio of the number of observed genes to the number of expected genes. The number of expected genes was calculated as N(set A) N(set B) / N(genome). The choice of N(genome) was based on the values recommended by the authors. For intersections with the Shapira et al. PA14 data set, N(genome) was set to 7308. For intersections with the Troemel et al. PA14 data set, N(genome) was set to 20000. N(genome) for the combined datasets was set to 20000. Intersection p-values were calculated from the hypergeometric distribution using R.

Genes that were concordantly up- or down-regulated were 2.0- and 2.4-fold more frequent

than chance, respectively ($p < 10^{-6}$ each). In contrast, genes that were upregulated in *daf-2* mutants and downregulated by PA14 were 3.7-fold enriched ($p < 10^{-21}$). Genes that were down in *daf-2* mutants and upregulated by PA14 were 6.7-fold enriched ($p < 10^{-85}$). When the analysis was repeated using a "narrow" inclusion criterion, only discordantly-regulated genes could be found (Table S6). The "narrow" PA14 dataset includes 96 induced and 11 repressed genes. The "narrow" *daf-2* dataset includes 123 induced and 62 repressed genes. No genes were upregulated in both *daf-2* mutants and during PA14 infection or downregulated in both *daf-2* mutants and during PA14 infection. However, 4 genes are up in *daf-2* mutants and down in response to PA14 (59 fold enrichment, $p < 10^{-6}$) and 11 genes are down in *daf-2* mutants and up in response to PA14 (37 fold enrichment, $p < 10^{-14}$). We also repeated the analysis with each source dataset disaggregated, which yielded similar results (Table S7). Thus, the observation that *daf-2* mutants and PA14 infection have predominately-discordant effects on gene expression is confirmed across a range of data sets using a variety of criteria for comparison.

To assess the functional significance of the discordant gene regulation, we asked whether genes required for *C. elegans* immunity are concordantly or discordantly regulated by loss of *daf-2* and PA14 infection. We first generated a list of 27 immune effector genes, which either have known antimicrobial function or demonstrated requirement for resistance to PA14 infection (Table S8). We then compared this list to *daf-2* and PA14 broad datasets. Among genes upregulated in *daf-2* mutants, 6 are in the immune effector gene set (3.6 fold enrichment, $p = 0.001$). Among genes downregulated in *daf-2* mutants, 6 are in the immune effector gene set (4.6 fold enrichment, $p < 0.001$). Among genes induced by PA14, 18 are in the immune effector gene set (27.6 fold enrichment, $p < 10^{-24}$). Among genes repressed by PA14, 6 are in the immune effector gene set (15.7 fold enrichment, $p < 10^{-7}$), thus confirming that some functionally-

significant infection response genes are repressed during PA14 infection, and suggesting that PA14 has the ability to repress immune function.

Among the intersections of “broad” *daf-2* and PA14 datasets, immune genes were found only in the discordant categories (Figure S10B). While zero immune genes were up or down in both *daf-2* mutants and in response to PA14, 3 immune genes are up in *daf-2* mutants and repressed in response to PA14 (33.2 fold enrichment, $p < 10^{-5}$), whereas 6 immune genes are down in *daf-2* mutants and induced in response to PA14 (28.9 fold enrichment, $p < 10^{-8}$). That a significant number of immune genes are regulated discordantly between *daf-2* mutants and PA14 infection raises the possibility that activation of DAF-2 mediates the downregulation a subset of immune genes during PA14 infection.

References

1. Shapira M, Hamlin BJ, Rong J, Chen K, Ronen M, et al. (2006) A conserved role for a GATA transcription factor in regulating epithelial innate immune responses. Proc Natl Acad Sci U S A 103: 14086-14091.
2. McElwee JJ, Schuster E, Blanc E, Thomas JH, Gems D (2004) Shared transcriptional signature in *Caenorhabditis elegans* Dauer larvae and long-lived *daf-2* mutants implicates detoxification system in longevity assurance. J Biol Chem 279: 44533-44543.
3. Murphy CT, McCarroll SA, Bargmann CI, Fraser A, Kamath RS, et al. (2003) Genes that act downstream of DAF-16 to influence the lifespan of *Caenorhabditis elegans*. Nature.
4. Troemel ER, Chu SW, Reinke V, Lee SS, Ausubel FM, et al. (2006) p38 MAPK regulates expression of immune response genes and contributes to longevity in *C. elegans*. PLoS Genet 2: e183.
