# Supplementary figures and images for: Pseudomonas aeruginosa Suppresses Host Immunity by Activating the DAF-2 Insulin-Like Signaling Pathway in Caenorhabditis elegans
Source: PLoS Pathog. 2008 Oct 17;4(10):e1000175. doi: 10.1371/journal.ppat.1000175 (PMC2568960; doi:10.1371/journal.ppat.1000175)

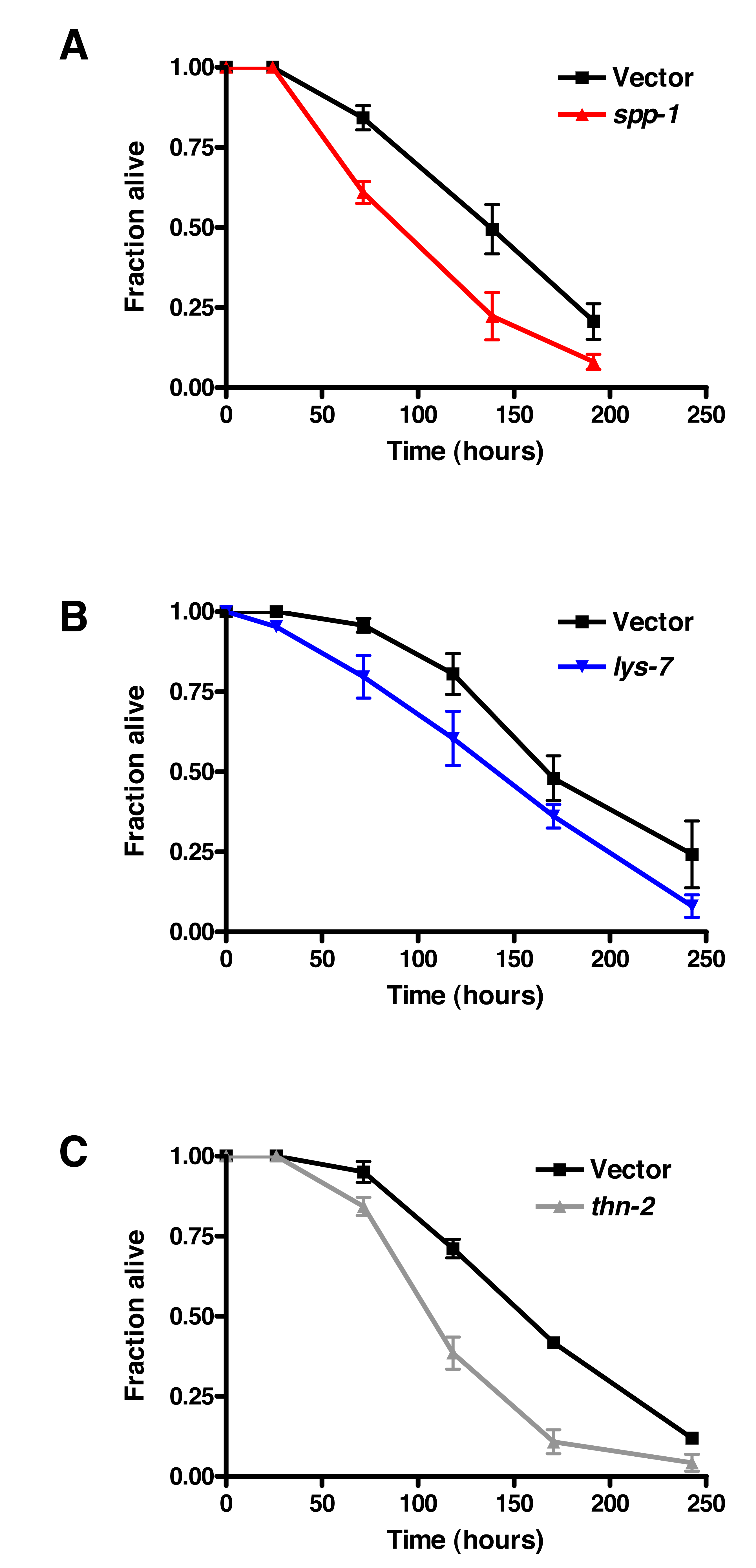

Supplement: Figure S1 — Knockdown of thn-2, lys-7, and spp-1 by RNAi enhances the susceptibility of C. elegans to P. aeruginosa infection. Survival of worms in which (A) spp-1, (B) lys-7, or (C) thn-2 was knocked down by RNAi followed by exposure to wildtype PA14 at 25°C was monitored over time. rrf-3(pk1426);glp-4(bn2) worms were used to enhance sensitivity to RNAi and to prevent progeny production, which can confound pathogen survival assays. (417 KB TIF) [file ppat.1000175.s002.tif]

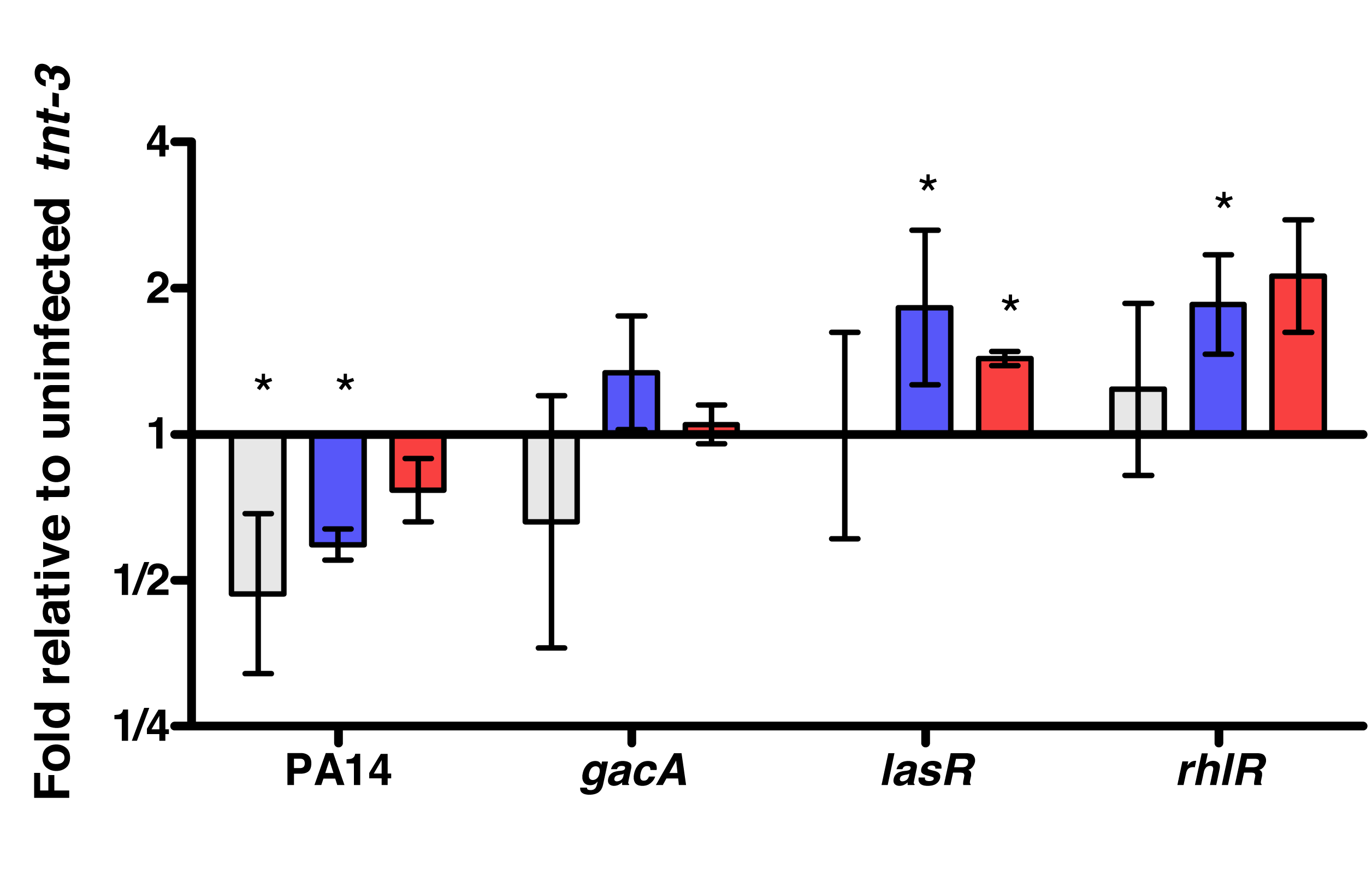

Supplement: Figure S2 — P. aeruginosa gacA, lasR, and rhlR are required immune suppression in C. elegans mutants that are unable to limit bacterial accumulation. Expression of downregulated host defense effectors genes in tnt-3(aj3) worms exposed to the PA14 and PA14 gacA, lasR, and rhlR mutants. Mean transcript levels are plotted relative to matched controls exposed to OP50-1. Error bars indicated SEM. At least 3 replicates of each condition were examined. * t-test, p<0.05 comparison to tnt-3(aj3) exposed to OP50-1. (166 KB TIF) [file ppat.1000175.s003.tif]

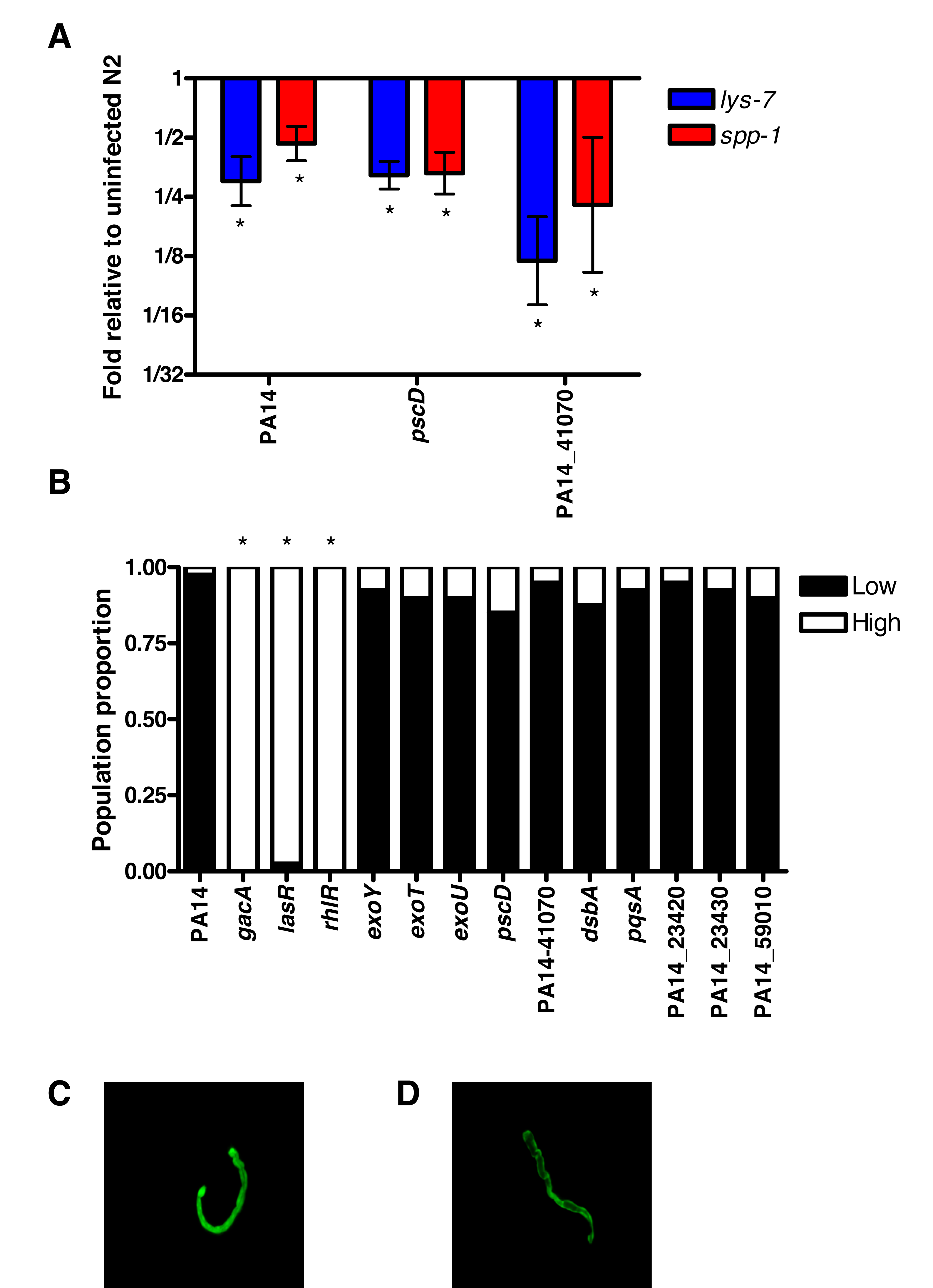

Supplement: Figure S3 — Many P. aeruginosa virulence factors are not required for immune suppression in C. elegans. (A) Expression of the host defense effector lys-7 and spp-1 in wildtype worms exposed to wildtype PA14 and the PA14 mutant pscD and PA14_41070 measured by qRT-PCR. Mean transcript levels are plotted relative to matched controls exposed to OP50-1. Error bars indicated SEM. 3 replicates of each condition were examined. * t-test, p<0.05 (B) Expression of the host defense effector lys-7 in wildtype worms exposed to wildtype PA14 and the PA14 mutants gacA, lasR, rhlR, exoU, exoT, exoY, pscD, PA14_41070, dsbA, pqsA, PA14_23420, PA14_23430, and PA14_59010 measured by visual classification of lys-7::GFP fluorescence as high or low intensity at 100× total magnification. Expression of lys-7::GFP in uninfected worms was the standard for high intensity fluorescence. Data is also shown in Table S1. * t-test, p<0.05 (C–D) Fluorescence micrographs of lys-7::GFP worms categorized as (C) high or (D) low expression. (442 KB TIF) [file ppat.1000175.s004.tif]

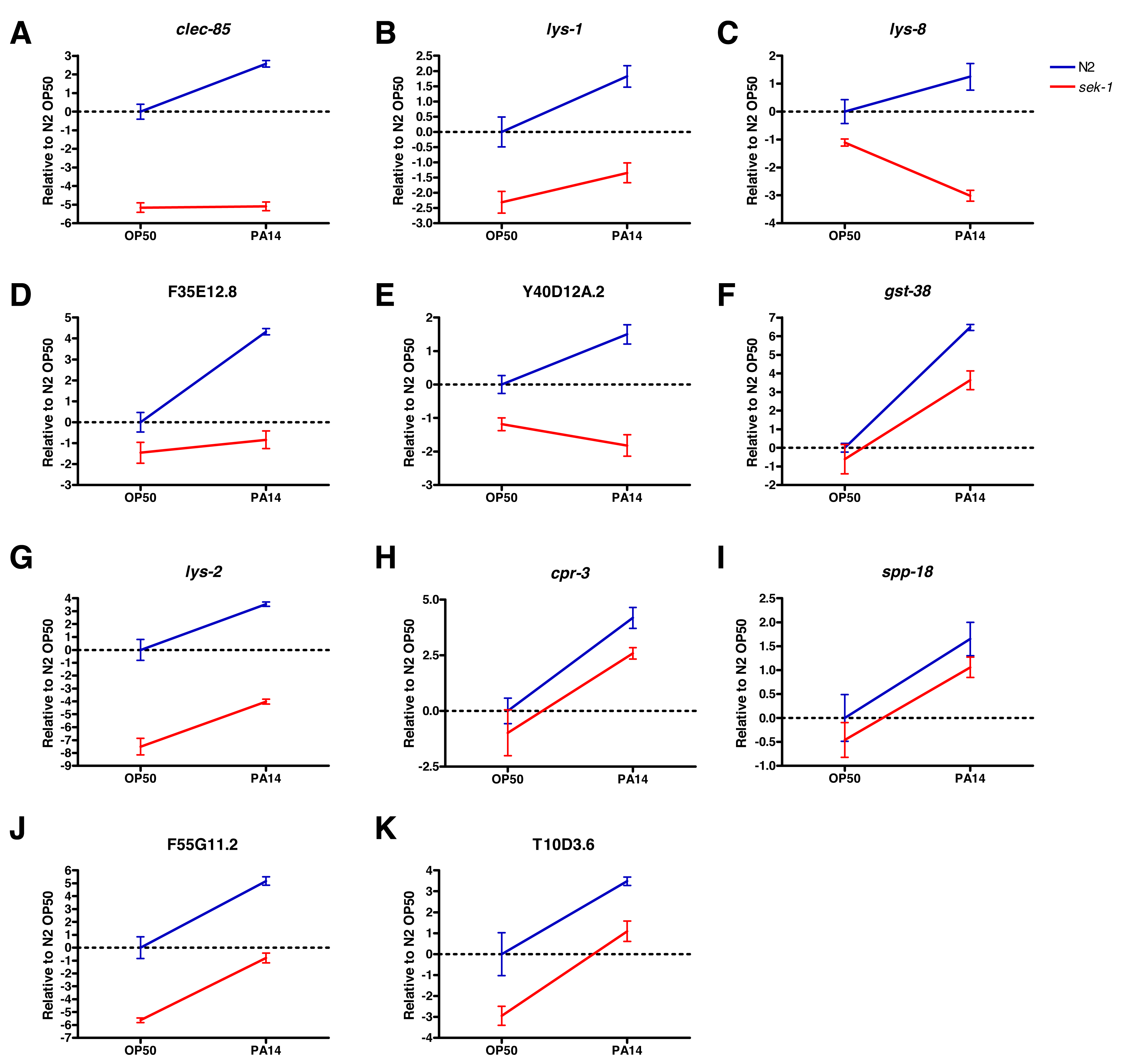

Supplement: Figure S4 — 2×2 factorial interaction plots depicting the contribution of p38 MAPK to the transcriptional response to P. aeruginosa. Both p38-dependent (A–F) and p38-independent (G–K) induction and repression of gene expression in response to PA14 infection are observed. Mean (and SEM) of log2 scale transcript levels relative to N2 OP50-1 were plotted for (A) clec-85, (B) lys-1, (C) lys-8, (D) F35E12.8, (E) Y40D12A.2, (F) gst-38, (G) lys-2, (H) cpr-3, (I) spp-18, (J) F55G11.2, (K) and T10D3.6 in N2 (blue) and sek-1(km4) (red) exposed to OP50-1 and PA14. (525 KB TIF) [file ppat.1000175.s005.tif]

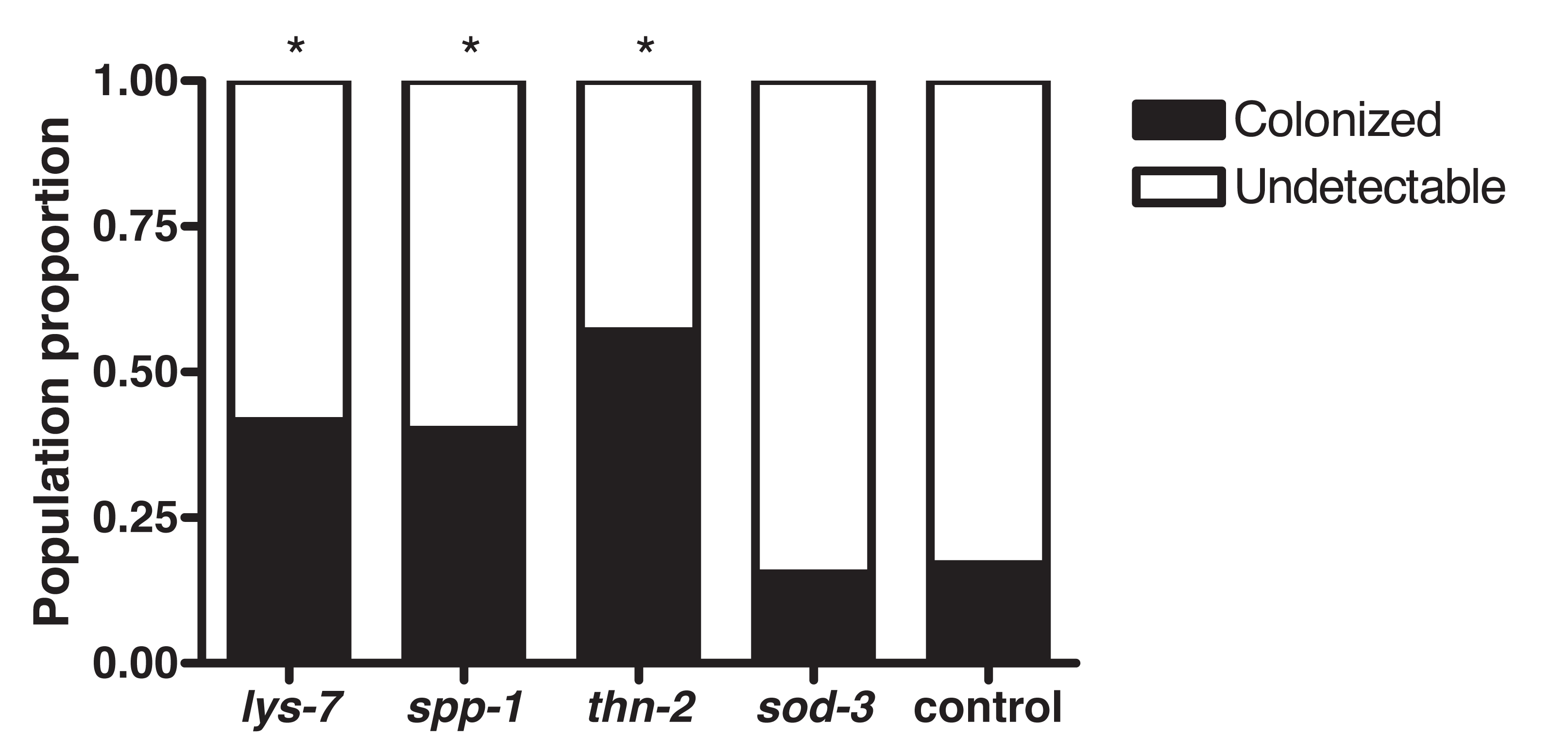

Supplement: Figure S5 — Knockdown of thn-2, lys-7, and spp-1 by RNAi decreases the resistance of daf-2(e1370) mutants to colonization by P. aeruginosa. RNAi knockdown of thn-2, lys-7, and spp-1 resulted in increased P. aeruginosa accumulation in daf-2(e1370) worms compared to control RNAi (Fisher's exact test; p = 0.0035, p = 0.0054, and p<0.0001, respectively). RNAi knockdown of sod-3 did not significantly affect colonization (Fisher's exact test, p>0.9999). RNAi or control treated daf-2(e1370) were exposed to a PA14 strain that expresses GFP (PA14-GFP) as young adults for 108 hr. Individual worms were classified as having detectable or undetectable GFP fluorescence in the intestinal lumen by visual inspection at 200× total magnification. For each RNAi treatment, a total of 65 worms were assayed blind. (193 KB TIF) [file ppat.1000175.s006.tif]

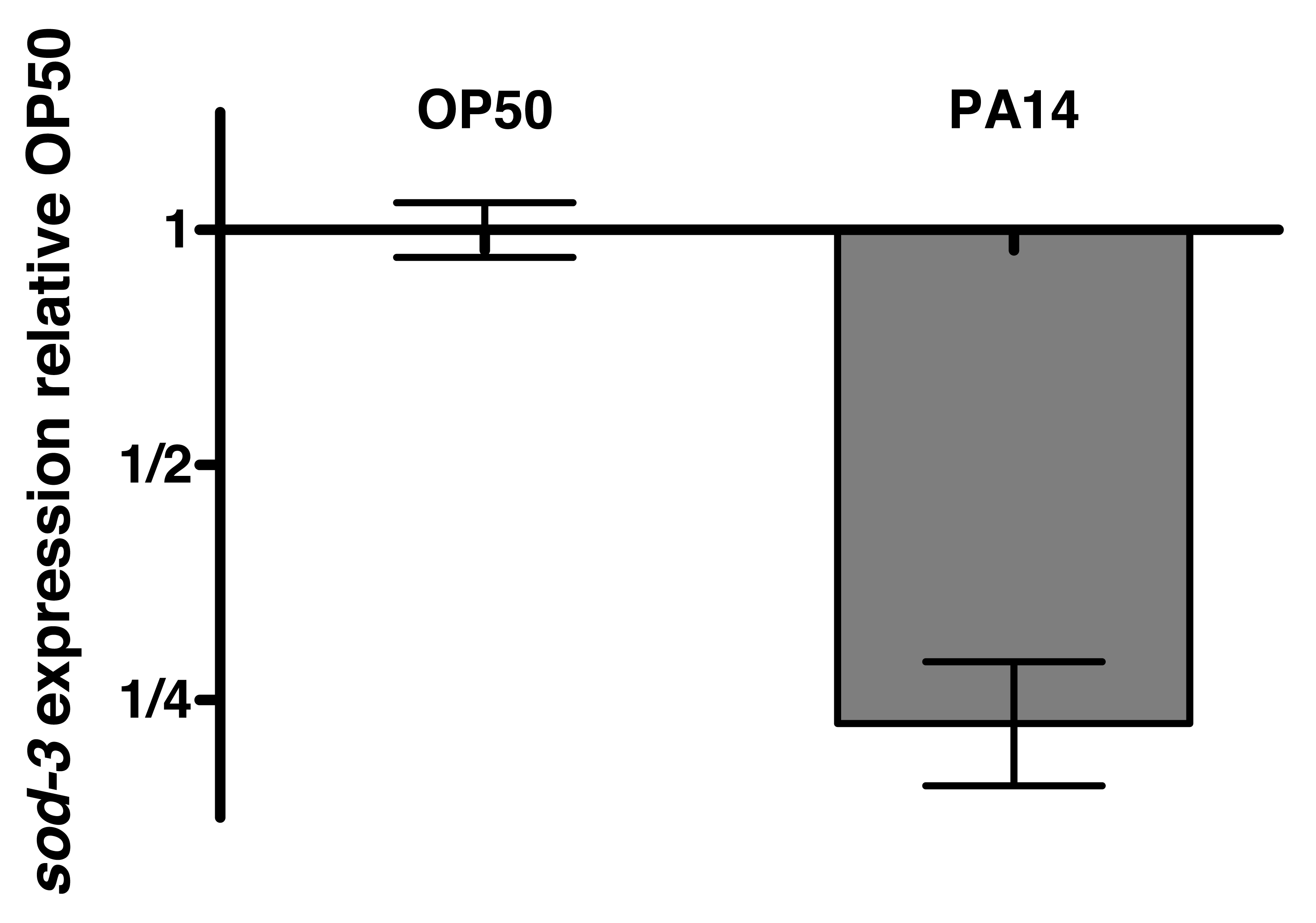

Supplement: Figure S6 — sod-3 expression is repressed following P. aeruginosa infection. qRT-PCR measurement of sod-3 transcript levels in N2 worms exposed to OP50-1 and PA14 is plotted relative to OP50-1 levels. * t-test, p<0.05. (734 KB TIF) [file ppat.1000175.s007.tif]

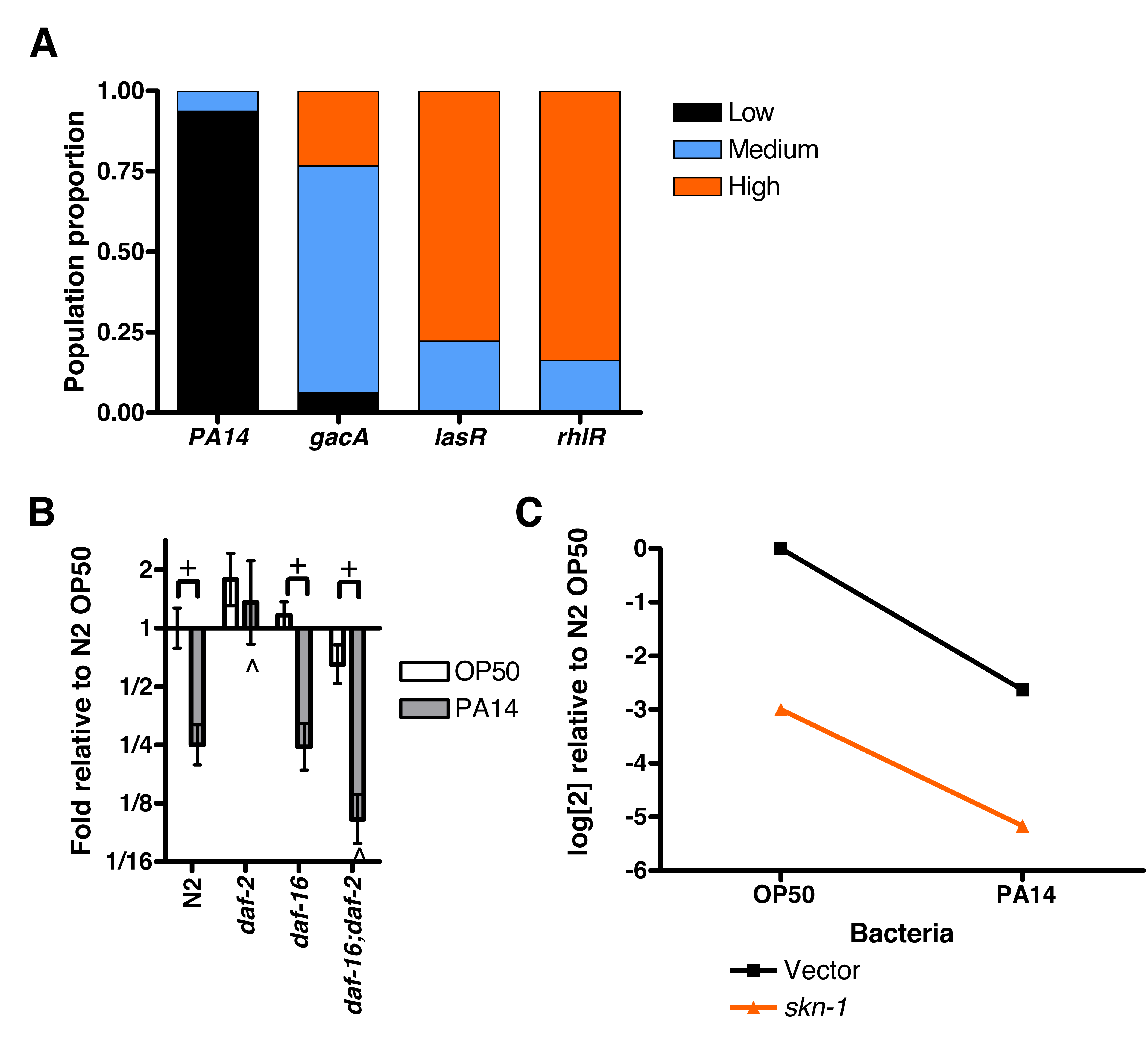

Supplement: Figure S7 — The expression of gst-4 is repressed in worms exposed to P. aeruginosa in a skn-1-independent manner. (A) Fluorescence intensity of gst-4::GFP in worms exposed to wildtype PA14 and the PA14 mutants gacA, lasR and rhlR. Worms of uniformly bright GFP intensity were placed on PA14 for 24 hours. Individual worms were scored for GFP intensity in three categories: low, medium, and high. Population proportions are significantly different in all pairwise comparisons with the exception of lasR and rhlR, which are statistically indistinguishable (Chi-square test, p<0.05). (B) Expression of gst-4 measured in N2, daf-2(e1370), daf-16(mu86), and daf-16(mu86);daf-2(e1370) exposed to OP50-1 and PA14. Mean transcript levels were plotted relative to N2 OP50-1. Error bars represent SEM. t-test ˆ p<0.05 comparison to N2 PA14, + p<0.05 comparing OP50-1 to PA14. (C) Mean (and SEM) of log2 scale transcript levels relative to N2 OP50-1 for gst-4 in skn-1 RNAi and vector control worms exposed to OP50-1 and PA14. (437 KB TIF) [file ppat.1000175.s008.tif]

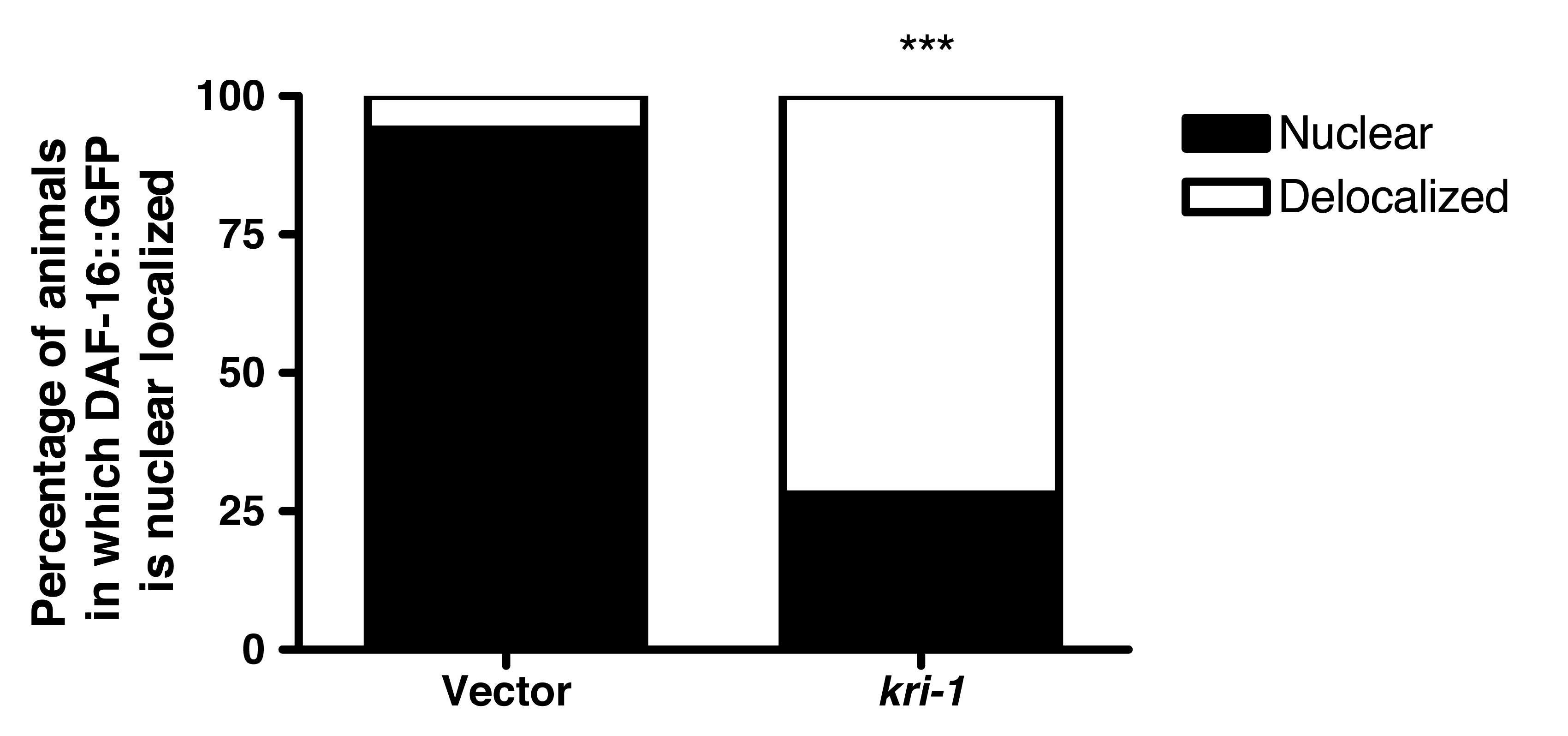

Supplement: Figure S8 — kri-1 is required for nuclear localization in cdc-25.1 RNAi-treated animals without proliferating germlines. The localization of DAF-16 in animals exposed first to cdc-25.1 RNAi and then kri-1 RNAi was assayed. *** Fisher's exact test, p<0.0001. (721 KB TIF) [file ppat.1000175.s009.tif]

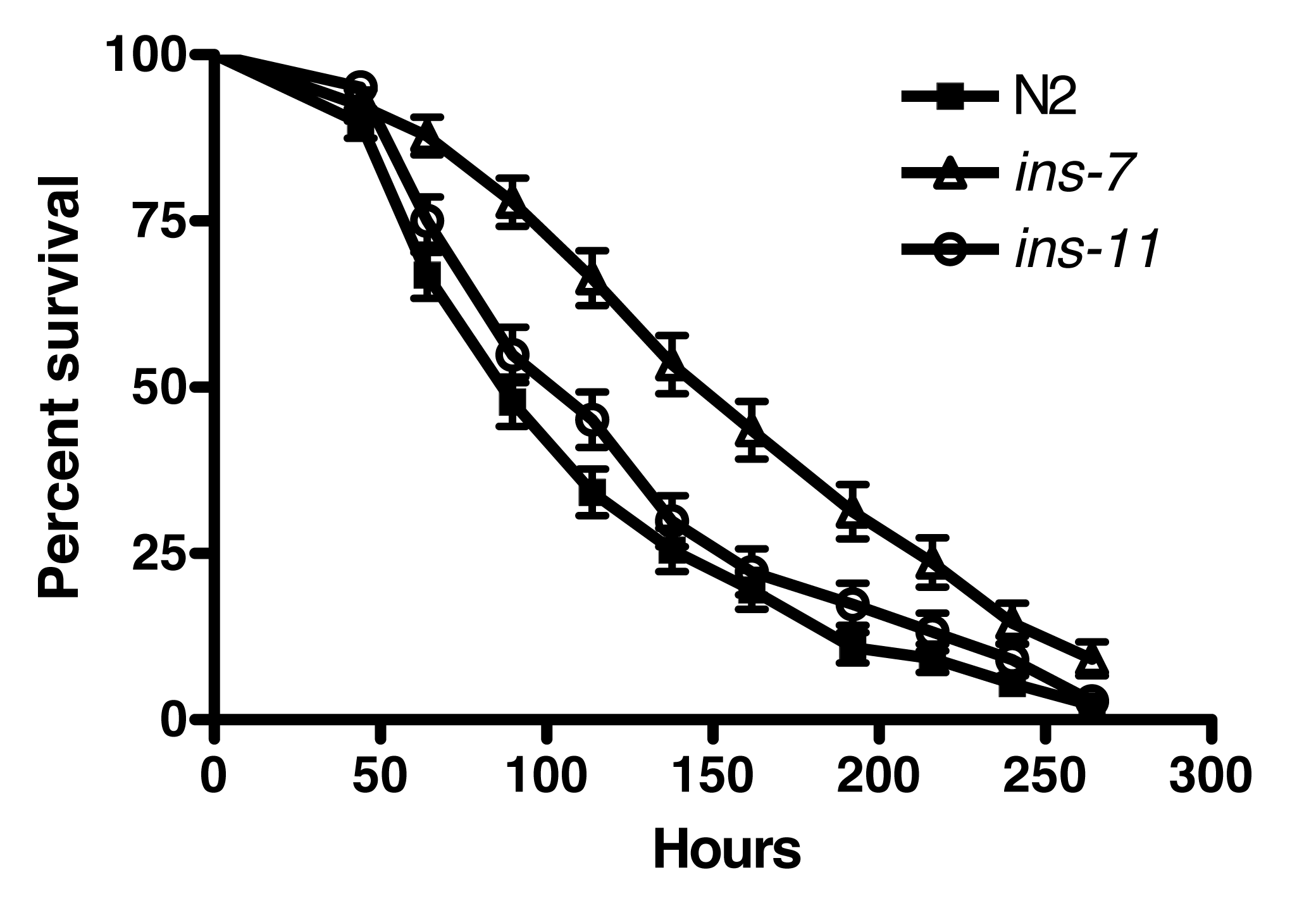

Supplement: Figure S9 — ins-7 but not ins-11 is required for resistance to P. aeruginosa. Survival of N2, ins-7(tm1907) and ins-11(tm1053) worms was monitored on PA14 over time at 25°C. ins-7(tm1907) is significantly more resistant to PA14 than N2 or ins-11(tm1053) (logrank, p<0.0001 and p = 0.0002, respectively). ins-11(tm1053) is statistically indistinguishable from N2 (logrank, p = 0.0902). (164 KB TIF) [file ppat.1000175.s010.tif]

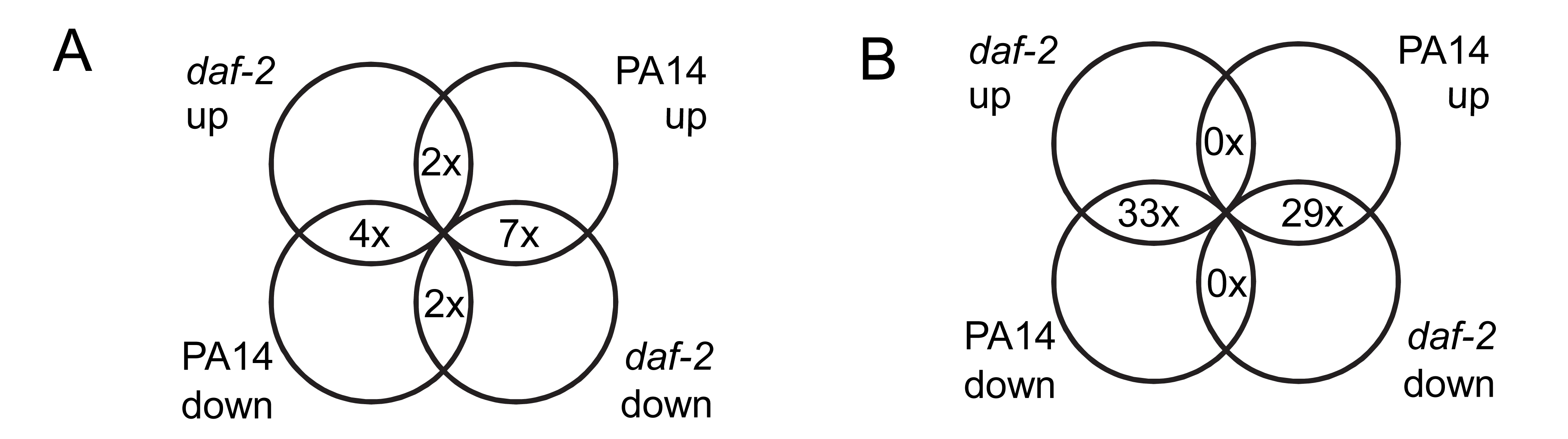

Supplement: Figure S10 — P. aeruginosa exposure negatively regulates host defense daf-2 target genes. (A) Venn diagram representing the intersection of the “broad” daf-2 and PA14 consensus datasets. Values are fold enrichment over chance. The greatest enrichment of genes that are affected in both daf-2 mutants and during PA14 infection was found in the inversely regulated categories. (B) Venn diagram representing the intersections of the “broad” daf-2 and PA14 datasets among immune genes. All immune genes that are affected in both daf-2 mutants and during PA14 infection are found in the inversely regulated categories. (236 KB TIF) [file ppat.1000175.s011.tif]
