# Supplementary material for: Pseudomonas aeruginosa Suppresses Host Immunity by Activating the DAF-2 Insulin-Like Signaling Pathway in Caenorhabditis elegans
Source: PLoS Pathog. 2008 Oct 17;4(10):e1000175. doi: 10.1371/journal.ppat.1000175 (PMC2568960; doi:10.1371/journal.ppat.1000175)
Supplement: Table S1 — Survival of pha-1(e2123) worms and lys-7::GFP expression in transgenic worms following exposure to PA14 and PA14 mutants. (6 KB PDF) [file ppat.1000175.s012.pdf]

Table S1. Survival of *pha-1(e2123)* worms and *lys-7::GFP* expression in transgenic worms following exposure to PA14 and PA14 mutants.

	Survival					*lys-7*::GFP[4]	
**Strain**	**Mean time to death (hr)**	**SEM**	**N dead[1]**	**N total[2]**	**Logrank p-value**	**Population proportion affected [3]**	**Fisher's exact, p-value**
PA14	54.5	1.1	220	241	-	0.963	-
Required for survival and immune suppression							
gacA	119.5	2.3	119	119	<0.0001	0.000	<0.0001
lasR	87.5	2.1	119	119	<0.0001	0.025	<0.0001
rhlR	99.6	2.6	118	119	<0.0001	0.000	<0.0001
Required for survival but not immune suppression							
PA14_41070	65.6	1.9	113	121	<0.0001	0.950	>0.9999
dsbA	66.9	2.3	118	118	<0.0001	0.875	0.1151
pqsA	65.1	1.8	117	120	<0.0001	0.925	0.399
PA14_23430	63.5	1.8	111	120	<0.0001	0.925	0.399
PA14_59010	68.2	1.6	122	123	<0.0001	0.900	0.2201
PA14_23420	71.7	2.1	122	109	<0.0001	0.950	>0.9999

[1] Number of deaths observed. [2] Total number of observations: N dead + N censored. [3] Proportion of population with reduced *lys-7::GFP* expression following exposure to pathogen. [4] Data also shown in Figure S3B.
