# Supplementary material for: Pseudomonas aeruginosa Suppresses Host Immunity by Activating the DAF-2 Insulin-Like Signaling Pathway in Caenorhabditis elegans
Source: PLoS Pathog. 2008 Oct 17;4(10):e1000175. doi: 10.1371/journal.ppat.1000175 (PMC2568960; doi:10.1371/journal.ppat.1000175)
Supplement: Table S3 — ins-7, but not ins-11, is required for resistance to PA14. Worms were sterilized by RNAi knockdown of cdc-25.1. Glp worms were exposed to PA14 and survival was monitored over time. (5 KB PDF) [file ppat.1000175.s014.pdf]

Table S3. *ins-7*, but not *ins-11*, is required for resistance to PA14

**Strain**	**Mean time to death (hr)**	**SEM**	**N dead[1]**	**N total[2]**	**P-value vs N2**	**P-value vs *ins-7***
N2	117.02	4.611	184	188	-	-
ins-7(tm1907)	162.36	6.055	131	143	<0.0001	-
ins-11(tm1053)	129.73	5.481	144	148	0.0902	0.0002

Worms were sterilized by RNAi knockdown of *cdc-25.1*. Glp worms were exposed to PA14 and survival was monitored over time. [1] Number of deaths observed. [2] Total number of observations: N dead + N censored.
