# Supplementary material for: Pseudomonas aeruginosa Suppresses Host Immunity by Activating the DAF-2 Insulin-Like Signaling Pathway in Caenorhabditis elegans
Source: PLoS Pathog. 2008 Oct 17;4(10):e1000175. doi: 10.1371/journal.ppat.1000175 (PMC2568960; doi:10.1371/journal.ppat.1000175)
Supplement: Table S4 — Intestinal-specific knockdown of daf-16 causes enhanced susceptibility to PA14. Worms were sterilized by 27.5°C treatment. (6 KB PDF) [file ppat.1000175.s015.pdf]

Table S4. Intestinal-specific knockdown of *daf-16* causes enhanced susceptibility to PA14

**Strain**	**RNAi**	**Mean time to death (hr)**	**SEM**	**N dead[1]**	**N total[2]**	**LogRank P-value (Strain)**	**LogRank P-value (RNAi)**
N2	control	45.598	2.015	77	77	-	-
N2	*daf-16*	43.945	1.492	74	82	-	0.4039
VP303	control	47.573	2.38	85	87	0.0738	-
VP303	*daf-16*	35.845	1.313	85	85	<0.0001	<0.0001
N2	control	55.993	1.667	106	113	-	-
N2	*daf-16*	55.636	1.736	103	109	-	0.8531
VP303	control	54.871	1.813	109	110	0.9428	-
VP303	*daf-16*	46.421	1.416	105	107	0.0002	0.0002
N2	control	83.509	2.209	101	133	-	-
N2	*daf-16*	84.045	1.998	108	127	-	0.8560
NR222	control	58.702	0.970	85	85	<0.0001	-
NR222	*daf-16*	56.363	1.299	70	70	<0.0001	0.1285
N2	control	56.36	1.7	107	114	-	-
N2	*unc-22*	56.39	1.9	72	76	-	0.8599
VP303	control	54.87	1.8	109	110	0.9488	-
VP303	*unc-22*	56.42	2.4	89	89	0.444	0.417

Worms were sterilized by 27.5°C treatment. [1] Number of deaths observed. [2] Total number of observations: N dead + N censored.
