# Supplementary material for: Pseudomonas aeruginosa Suppresses Host Immunity by Activating the DAF-2 Insulin-Like Signaling Pathway in Caenorhabditis elegans
Source: PLoS Pathog. 2008 Oct 17;4(10):e1000175. doi: 10.1371/journal.ppat.1000175 (PMC2568960; doi:10.1371/journal.ppat.1000175)
Supplement: Table S5 — Strains. (20 KB PDF) [file ppat.1000175.s016.pdf]

Table S5. Strains

**Strain**	**Description**	**Source or reference**
**Bacterial Strains**		
OP50-1	A streptomycin-resistant derivative of *Escherichia coli* OP50 used as food source in *C. elegans* cultivation and as uninfected control	*Caenorhabditis* genetics center (CGC)
PA14	Virulent clinical isolate of *P. aeruginosa* from burn wound	[1]
PA14-GFP	Isogenic GFP-expressing strain of PA14	[2]
SL1344	Wildtype *S. enterica* serovar. Typhimurium	D. Monack (Stanford University)
V583	Wildtype *Enterococcus faecalis*	[3]
PA14 *gacA*	*gacA::Kan* nonpolar	[2]
PA14 *lasR*	*lasR::TnphoA*	[4]
PA14 *rhlR*	*rhlR::MAR2xT7*	[5]
PA14_41070	*PA14_41070::MAR2xT7*, putative S-adenosyl-methionine-dependent methyltransferase similar to PA1814	[5]
PA14 *dsbA*	*dsbA::MAR2xT7*	[5]
PA14 *pqsA*	*pqsA::MAR2xT7*	[5]
PA14_23430	PA14_23430*::MAR2xT7*	[5]
PA14_59010	PA14_59010*::MAR2xT7*	[5]
PA14_23420	PA14_23420*::MAR2xT7*	[5]
PA14 Δ*pscD*	1.17-kb in-frame deletion of *pscD* and nonpolar on the expression of downstream genes	[6]
PA14 Δ*exoU*	2.0-kb deletion of *exoU*	[6]
PA14 Δ*exoY*	1.1-kb deletion of *exoY*	[6]
PA14 Δ*exoT*	1.3-kb in-frame deletion of *exoT*	[6]
**Worm Strains**		
N2	Wildtype *Caenorhabditis elegans*	CGC [7]
tnt-3(aj3)	Also called *esp-1(aj3)*	[8]
daf-2(e1370)	CB1370, loss-of-function missense mutation in the kinase domain of *daf-2*	CGC
daf-16(mu86)	CF1038, deletion of *daf-16*	CGC
daf-16(mu86); *daf-2(e1370)*	CF1515, non-roller non-GFP line selected	CGC
sek-1(km4)	KU4, deletion of *sek-1*	CGC
sma-6(wk7)	LT186, early stop (Y72X) mutation in *sma-6*	CGC
DAF-16::GFP	TJ356, integrated DAF-16::GFP roller strain	CGC [9]
ins-7(tm1970)	A 485-bp deletion mutant of *ins-7*, which was outcrossed 4 times.	National BioResource Project, Japan
ins-11(tm1053)	A 341-bp deletion mutant of *ins-11*, which was outcrossed 4 times.	National BioResource Project, Japan
VP303	*rde-1(ne219)*; *kbEx200* [*rol-6(su1006)*;p*nhx-2*::RDE-1]	K. Strange (Vanderbilt University) [10]
NR222	*rde-1(ne219)*; kzIs9 [pKK1260(*lin-26*p::NLS::GFP), pKK1253(*lin-26*p::RDE-1), pRF4(*rol-6(su1006)*)]);	K. Strange (Vanderbilt University) [11]
rrf-3(pk1426); glp-4(bn2)	Double mutant carrying loss-of-function mutations in *rrf-3* and *glp-4*	[12]
pha-1(e2123)	Unknown loss-of-function mutation in *pha-1*	CGC
lys-7::GFP	A transcriptional fusion of the *lys-7* promoter to GFP	S. Alper [13]
gst-4::GFP	A transcriptional fusion of the *gst-4* promoter to GFP	CGC

References

1. Rahme LG, Stevens EJ, Wolfort SF, Shao J, Tompkins RG, et al. (1995) Common virulence factors for bacterial pathogenicity in plants and animals. Science 268: 1899-1902.

2. Tan MW, Mahajan-Miklos S, Ausubel FM (1999) Killing of *Caenorhabditis elegans* by *Pseudomonas aeruginosa* used to model mammalian bacterial pathogenesis. Proc Natl Acad Sci U S A 96: 715-720.

3. Sahm DF, Kissinger J, Gilmore MS, Murray PR, Mulder R, et al. (1989) In vitro susceptibility studies of vancomycin-resistant *Enterococcus faecalis*. Antimicrob Agents Chemother 33: 1588-1591.

4. Tan MW, Rahme LG, Sternberg JA, Tompkins RG, Ausubel FM (1999) *Pseudomonas aeruginosa* killing of *Caenorhabditis elegans* used to identify *P. aeruginosa* virulence factors. Proc Natl Acad Sci U S A 96: 2408-2413.

5. Liberati NT, Urbach JM, Miyata S, Lee DG, Drenkard E, et al. (2006) An ordered, nonredundant library of *Pseudomonas aeruginosa* strain PA14 transposon insertion mutants. Proc Natl Acad Sci U S A 103: 2833-2838.

6. Miyata S, Casey M, Frank DW, Ausubel FM, Drenkard E (2003) Use of the Galleria mellonella caterpillar as a model host to study the role of the type III secretion system in *Pseudomonas aeruginosa* pathogenesis. Infect Immun 71: 2404-2413.

7. Brenner S (1974) The genetics of *Caenorhabditis elegans*. Genetics 77: 71-94.

8. Kim DH, Feinbaum R, Alloing G, Emerson FE, Garsin DA, et al. (2002) A conserved p38 MAP kinase pathway in *Caenorhabditis elegans* innate immunity. Science 297: 623-626.

9. Henderson ST, Johnson TE (2001) *daf-16* integrates developmental and environmental inputs to mediate aging in the nematode *Caenorhabditis elegans*. Curr Biol 11: 1975-1980.

10. Espelt MV, Estevez AY, Yin X, Strange K (2005) Oscillatory Ca2+ signaling in the isolated *Caenorhabditis elegans* intestine: role of the inositol-1,4,5-trisphosphate receptor and phospholipases C beta and gamma. J Gen Physiol 126: 379-392.

11. Qadota H, Inoue M, Hikita T, Koppen M, Hardin JD, et al. (2007) Establishment of a tissue-specific RNAi system in *C. elegans*. Gene 400: 166-173.

12. Shapira M, Hamlin BJ, Rong J, Chen K, Ronen M, et al. (2006) A conserved role for a GATA transcription factor in regulating epithelial innate immune responses. Proc Natl Acad Sci U S A 103: 14086-14091.

13. Alper S, McBride SJ, Lackford B, Freedman JH, Schwartz DA (2007) Specificity and complexity of the *Caenorhabditis elegans* innate immune response. Mol Cell Biol 27: 5544-5553.
