# Supplementary material for: Pseudomonas aeruginosa Suppresses Host Immunity by Activating the DAF-2 Insulin-Like Signaling Pathway in Caenorhabditis elegans
Source: PLoS Pathog. 2008 Oct 17;4(10):e1000175. doi: 10.1371/journal.ppat.1000175 (PMC2568960; doi:10.1371/journal.ppat.1000175)
Supplement: Table S7 — DAF-2-regulated genes are enriched for discordantly regulated infection-response genes. (6 KB PDF) [file ppat.1000175.s018.pdf]

Table S7. DAF-2-regulated genes are enriched for discordantly regulated infection-response genes.

		Shapira[1]		Troemel[2] 4h		Troemel[2] 8h	
		Up (n = 196)	Down (n = 34)	Up (n = 309)	Down (n = 121)	Up (n = 270)	Down (n = 232)
Murphy [3]	Up	1 (p = 0.4)	**10.9** ($p < 10^{-11}$)	**4.5** ($p < 10^{-7}$)	**8.4** ($p < 10^{-9}$)	**4.9** ($p < 10^{-7}$)	**8.1** ($p < 10^{-15}$)
	Down	**4.1** ($p < 10^{-10}$)	0 (p = 0.7)	**6.8** ($p < 10^{-14}$)	1.3 (p = 0.2)	**6.9** ($p < 10^{-13}$)	1.7 (p = 0.1)
McElwee [4]	Up	**0.4** ($p < 10^{-4}$)	1.2 (p < 0.2)	**1.9** ($p < 10^{-3}$)	**4.2** ($p < 10^{-10}$)	1.2 (p = 0.2)	**3.8** ($p < 10^{-16}$)
	Down	**2.6** ($p < 10^{-11}$)	0.3 (p < 0.9)	**9.5** ($p < 10^{-81}$)	**4.2** ($p < 10^{-8}$)	**10** ($p < 10^{-77}$)	**2.7** ($p < 10^{-5}$)

Values are fold enrichment of the intersection of each set relative to the expected intersection. P-values are calculated based on the hypergeometric distribution.

References

1. Shapira M, Hamlin BJ, Rong J, Chen K, Ronen M, et al. (2006) A conserved role for a GATA transcription factor in regulating epithelial innate immune responses. Proc Natl Acad Sci U S A 103: 14086-14091.
2. Troemel ER, Chu SW, Reinke V, Lee SS, Ausubel FM, et al. (2006) p38 MAPK regulates expression of immune response genes and contributes to longevity in *C. elegans*. PLoS Genet 2: e183.
3. Murphy CT, McCarroll SA, Bargmann CI, Fraser A, Kamath RS, et al. (2003) Genes that act downstream of DAF-16 to influence the lifespan of *Caenorhabditis elegans*. Nature.
4. McElwee JJ, Schuster E, Blanc E, Thomas JH, Gems D (2004) Shared transcriptional signature in *Caenorhabditis elegans* Dauer larvae and long-lived *daf-2* mutants implicates detoxification system in longevity assurance. J Biol Chem 279: 44533-44543.
