# Supplementary material for: Pseudomonas aeruginosa Suppresses Host Immunity by Activating the DAF-2 Insulin-Like Signaling Pathway in Caenorhabditis elegans
Source: PLoS Pathog. 2008 Oct 17;4(10):e1000175. doi: 10.1371/journal.ppat.1000175 (PMC2568960; doi:10.1371/journal.ppat.1000175)
Supplement: Table S8 — List of immune effector genes: genes encoding proteins with antimicrobial activity and/or required to protect C. elegans from P. aeruginosa infection. (17 KB PDF) [file ppat.1000175.s019.pdf]

Table S8. List of immune effector genes: genes encoding proteins with antimicrobial activity and/or required to protect *C. elegans* from *P. aeruginosa* infection.

Gene	Description	Evidence	Survival on PA14 (relative to N2)
abf-2	Antimicrobial peptide similar to ASABF (*Ascaris suum* antibacterial factor)	D	nt
acdh-1	Mitochondrial short-chain acyl-CoA dehydrogenase	A	0.78
asp-3	Aspartyl protease	B	0.77
C49C3.9	Intestinally-enriched expression	A	0.77
C55A6.7	Predicted short chain-type dehydrogenase	A	0.69
col-179	Collagen, possible signaling component	A	0.85
dct-17	Contains CUB-like domain	C	0.80
F08G5.6	Contains CUB-like domain	A	0.74
F20G2.5	Contains CUB-like domain	A	0.88
F41C3.8	Neuronal, pharyngeal, and intestinal expression	A	0.82
F53C11.3	Predicted short-chain dehydrogenase/reductase	A	0.68
gst-1	Putative glutathione S-transferase	A	0.86
his-16	H2A histone	A	0.62
K11D12.5	Putative multitransmembrane protein; intestinally-enriched expression	A	0.87
lys-2	Lysozyme	B	0.82
lys-7	Lysozyme	B	0.72
M60.1/2	Intestinally-enriched expression	A	0.64
maoc-1	Predicted to function in peroxisomal fatty acid beta-oxidation	A	0.80
nhr-112	Nuclear hormone receptor	A	0.87
pqm-1	Putative oxidative-stress responsive transcription factor	A	0.57
prx-11	Peroxin homolog; putative peroxisomal biogenesis function	A	0.70
skr-3	Ancient, conserved protein, putative skp1-related ubiquitin ligase	A	0.83
spp-1	Saposin-like antimicrobial similar to the amoebapores of *Entamoeba histolytica*	B,E	0.82
T19D12.4	Contains domain similar to von Willebrand factor type A	A	0.83
thn-1	Similar to thaumatin family of plant pathogenesis proteins	A	0.87
thn-2	Similar to thaumatin family of plant pathogenesis proteins	B	0.76
vab-9	Claudin homolog, role in epithelial development and morphology	A	0.83
Evidence codes: A = RNAi knockdown increases sensitivity to PA14 [1]; B = RNAi knockdown increases sensitivity to PA14 (unpublished data); C = RNAi knockdown increases sensitivity to PA14 (M. Nandakumar and M.-W. Tan, unpublished data); D = Antimicrobial activity [2]; E = Antimicrobial activity [3]. nt = not tested for PA14, required for resistance to colonization by *S. typhimurium*			

References

1. Shapira M, Hamlin BJ, Rong J, Chen K, Ronen M, et al. (2006) A conserved role for a GATA transcription factor in regulating epithelial innate immune responses. Proc Natl Acad Sci U S A 103: 14086-14091.

2. Kato Y, Aizawa T, Hoshino H, Kawano K, Nitta K, et al. (2002) *abf-1* and *abf-2*, ASABF-

type antimicrobial peptide genes in *Caenorhabditis elegans*. Biochem J 361: 221-230.
3. Banyai L, Patthy L (1998) Amoebapore homologs of *Caenorhabditis elegans*. Biochim Biophys Acta 1429: 259-264.
